# Supplementary material for: Numbers and types of neurological emergencies in England and the influence of socioeconomic deprivation: a retrospective analysis of hospital episode statistics data
Source: BMJ Open. 2022 Nov 4;12(11):e061843. doi: 10.1136/bmjopen-2022-061843 (PMC9639083; doi:10.1136/bmjopen-2022-061843)
Supplement: Supplementary data [file bmjopen-2022-061843supp001.pdf]

Appendix 1

Neurological Emergency ICD10 codes – 96 codes (short list)

**NeuroEmergencyConditionGroup**	**NeuroEmergencyICD10CodeHES**	**ICD10_Level3Header**
Acute paralysis	A521	Symptomatic neurosyphilis
Encephalitis	A830	Japanese encephalitis
Encephalitis	A831	Western equine encephalitis
Encephalitis	A832	Eastern equine encephalitis
Encephalitis	A833	St Louis encephalitis
Encephalitis	A834	Australian encephalitis
Encephalitis	A835	California encephalitis
Encephalitis	A836	Rocio virus disease
Encephalitis	A838	Other mosquito-borne viral encephalitis
Encephalitis	A839	Mosquito-borne viral encephalitis
Encephalitis	A840	Far Eastern tick-borne encephalitis [Russian spring-summer encephalitis]
Encephalitis	A841	Central European tick-borne encephalitis
Encephalitis	A848	Other tick-borne viral encephalitis
Encephalitis	A849	Tick-borne viral encephalitis
Encephalitis	A850	Enteroviral encephalitis
Encephalitis	A851	Adenoviral encephalitis
Encephalitis	A852	Arthropod-borne viral encephalitis, unspecified
Encephalitis	A858	Other specified viral encephalitis

Encephalitis	A86X	Unspecified viral encephalitis
Functional Disorders	F440	Dissociative amnesia
Functional Disorders	F441	Dissociative fugue
Functional Disorders	F442	Dissociative stupor
Functional Disorders	F443	Trance and possession disorders
Functional Disorders	F444	Dissociative motor disorders
Functional Disorders	F445	Dissociative convulsions
Functional Disorders	F446	Dissociative anaesthesia and sensory loss
Functional Disorders	F447	Mixed dissociative conversion disorders
Functional Disorders	F448	Other dissociative conversion disorders
Functional Disorders	F449	Dissociative conversion disorder unspecified
Encephalitis	G040	Acute disseminated encephalitis
Encephalitis	G041	Tropical spastic paraplegia
Encephalitis	G042	Bacterial meningoencephalitis and meningomyelitis NEC
Encephalitis	G048	Other encephalitis myelitis and encephalomyelitis
Encephalitis	G049	Encephalitis myelitis and encephalomyelitis unspecified
Encephalitis	G050	Encephalitis myelitis and encephalomyelitis in bacterial diseases classified elsewhere
Encephalitis	G051	Encephalitis myelitis and encephalomyelitis in viral diseases classified elsewhere
Encephalitis	G052	Encephalitis myelitis and encephalomyelitis in other infectious and parasitic diseases classified elsewhere
Encephalitis	G058	Encephalitis myelitis and encephalomyelitis in other diseases classified elsewhere
Acute nerve root/cord syndrome	G114	Hereditary spastic paraplegia
Epilepsy	G409	Epilepsy unspecified
Epilepsy	G410	Grand mal status epilepticus
Epilepsy	G411	Petit mal status epilepticus

Epilepsy	G412	Complex partial status epilepticus
Epilepsy	G418	Other status epilepticus
Epilepsy	G419	Status epilepticus unspecified
Acute nerve root/cord syndrome	G549	Nerve root and plexus disorder
Acute nerve root/cord syndrome	G551	Nerve root and plexus compressions in intervertebral disc disorders
Acute paralysis	G610	Guillain-Barre syndrome
Acute nerve root/cord syndrome	G820	Flaccid paraplegia
Acute nerve root/cord syndrome	G821	Spastic paraplegia
Acute nerve root/cord syndrome	G822	Paraplegia unspecified
Acute nerve root/cord syndrome	G823	Flaccid tetraplegia
Acute nerve root/cord syndrome	G824	Spastic tetraplegia
Acute nerve root/cord syndrome	G825	Tetraplegia unspecified
Acute nerve root/cord syndrome	G952	Cord compression unspecified
Acute nerve root/cord syndrome	G992	Myelopathy in diseases classified elsewhere
Subarachnoid haemorrhage	I600	Subarachnoid haemorrhage from carotid siphon and bifurcation
Subarachnoid haemorrhage	I601	Subarachnoid haemorrhage from middle cerebral artery

Subarachnoid haemorrhage	I602	Subarachnoid haemorrhage from anterior communicating artery
Subarachnoid haemorrhage	I603	Subarachnoid haemorrhage from posterior communicating artery
Subarachnoid haemorrhage	I604	Subarachnoid haemorrhage from basilar artery
Subarachnoid haemorrhage	I605	Subarachnoid haemorrhage from vertebral artery
Subarachnoid haemorrhage	I606	Subarachnoid haemorrhage from other intracranial arteries
Subarachnoid haemorrhage	I607	Subarachnoid haemorrhage from intracranial artery, unspecified
Subarachnoid haemorrhage	I608	Other subarachnoid haemorrhage
Subarachnoid haemorrhage	I609	Subarachnoid haemorrhage, unspecified
Subarachnoid haemorrhage	I690	Sequelae of subarachnoid haemorrhage
Acute nerve root/cord syndrome	M470	Anterior spinal and vertebral artery compression syndromes
Acute nerve root/cord syndrome	M471	Other spondylosis with myelopathy
Acute nerve root/cord syndrome	M490	Tuberculosis of spine
Acute nerve root/cord syndrome	M500	Cervical disc disorder with myelopathy - linked to G992A
Acute nerve root/cord syndrome	M501	Cervical disc disorder with radiculopathy
Acute nerve root/cord syndrome	M510	Lumbar and other intervertebral disc disorders with myelopathy
Acute nerve root/cord syndrome	M511	M51.1(dagger) Lumbar and other intervertebral disc disorders with radiculopathy (G55.1(asterix))
Acute nerve root/cord syndrome	M612	Paralytic calcification and ossification of muscle (Myositis ossificans associated with quadriplegia or paraplegia)
Head Injuries	S040	Injury of optic nerve and pathways

Head Injuries	S041	Injury of oculomotor nerve
Head Injuries	S042	Injury of trochlear nerve
Head Injuries	S043	Injury of trigeminal nerve
Head Injuries	S044	Injury of abducent nerve
Head Injuries	S045	Injury of facial nerve
Head Injuries	S046	Injury of acoustic nerve
Head Injuries	S047	Injury of accessory nerve
Head Injuries	S048	Injury of other cranial nerves
Head Injuries	S049	Injury of unspecified cranial nerve
Head Injuries	S060	Concussion
Head Injuries	S061	Traumatic cerebral oedema
Head Injuries	S062	Diffuse brain injury
Head Injuries	S063	Focal brain injury
Head Injuries	S064	Epidural haemorrhage
Head Injuries	S065	Traumatic subdural haemorrhage
Subarachnoid haemorrhage	S066	Traumatic subarachnoid haemorrhage
Head Injuries	S067	Intracranial injury with prolonged coma
Head Injuries	S068	Other intracranial injuries
Head Injuries	S069	Intracranial injury unspecified
Acute nerve root/cord syndrome	T093	Injury of spinal cord level unspecified
