# Supplementary material for: Numbers and types of neurological emergencies in England and the influence of socioeconomic deprivation: a retrospective analysis of hospital episode statistics data
Source: BMJ Open. 2022 Nov 4;12(11):e061843. doi: 10.1136/bmjopen-2022-061843 (PMC9639083; doi:10.1136/bmjopen-2022-061843)
Supplement: Supplementary data [file bmjopen-2022-061843supp002.pdf]

Supplementary Table 1: Percentage of in-year emergency hospital admissions with mention of neurological emergency codes admitted under the care of a consultant neurologist or neurosurgeon; adults aged 18 years and over; England; 2019/20

	Neurological Emergency	With Acute Nerve Root Mention	With Acute Paralysis Mention	With Encephalitis Mention	With Epilepsy Mention	With Functional Disorders Mention	With Head Injuries Mention	With Subarachnoid Haemorrhage Mention
admissions under neurology consultant	3,230	545	145	270	1,315	445	305	440
%	*1.36%*	*1.32%*	*5.58%*	*5.19%*	*0.90%*	*6.80%*	*1.04%*	*2.38%*
admissions under neurosurgery consultant	10,760	2,905	10	255	810	40	4,360	4,375
%	*4.52%*	*7.05%*	*0.42%*	*4.89%*	*0.56%*	*0.63%*	*14.91%*	*23.63%*
admissions under neurology or neurosurgery consultant	13,860	3,355	155	525	2,120	485	4,655	4,800
%	*5.83%*	*8.15%*	*6.00%*	*10.06%*	*1.45%*	*7.40%*	*15.92%*	*25.95%*
admissions with neurology consultant at a later point	3,150	435	250	410	1,355	370	390	320

%	*1.32%*	*1.06%*	*9.58%*	*7.81%*	*0.93%*	*5.67%*	*1.33%*	*1.72%*
admissions with neurosurgery consultant at a later point	2,265	795	*	55	165	10	945	835
%	*0.95%*	*1.93%*	*0.00%*	*1.07%*	*0.11%*	*0.17%*	*3.23%*	*4.52%*
admissions with neurology or neurosurgery consultant at a later point	5,000	1,110	245	430	1,465	375	1,200	1,010
%	*2.10%*	*2.70%*	*9.35%*	*8.21%*	*1.00%*	*5.72%*	*4.11%*	*5.49%*
admissions with no record of neurology or neurosurgery consultant	218,890	36,745	2,200	4,280	142,410	5,690	23,380	12,690
%	*92.07%*	*89.15%*	*84.64%*	*81.73%*	*97.54%*	*86.89%*	*79.97%*	*68.56%*
**in year admissions**	**237,755**	**41,215**	**2,600**	**5,240**	**145,995**	**6,545**	**29,235**	**18,505**
Source: OHID Neurology Dementia Intelligence using Hospital Episode Statistics Admited Patient Care, NHS Digital								

Supplementary Table 2

Deciles of Deprivation England		All HESIDs	With Epilepsy	With Functional Disorders	With Subarachnoid Haemorrhage
**1 Most deprived**	Count	23,600	14,970	910	1,770
	Percentage	14.5%	16.8%	19.5%	11.0%
**2**	Count	19,965	11,990	700	1,640
	Percentage	12.3%	13.4%	15.0%	10.2%
**3**	Count	18,365	10,595	555	1,640
	Percentage	11.3%	11.9%	11.8%	10.2%
**4**	Count	17,175	9,640	500	1,650
	Percentage	10.6%	10.8%	10.7%	10.3%
**5**	Count	16,005	8,700	465	1,590
	Percentage	9.9%	9.8%	9.9%	9.9%
**6**	Count	15,190	7,920	375	1,655
	Percentage	9.4%	8.9%	8.1%	10.3%
**7**	Count	14,340	7,365	340	1,525
	Percentage	8.8%	8.3%	7.3%	9.5%
**8**	Count	13,510	6,700	315	1,505
	Percentage	8.3%	7.5%	6.7%	9.4%
**9**	Count	12,920	6,080	300	1,605
	Percentage	8.0%	6.8%	6.3%	10.0%
**10 Least deprived**	Count	11,375	5,225	220	1,470
	Percentage	7.0%	5.9%	4.7%	9.2%

Source: OHID Neurology Dementia Intelligence using Hospital Episode Statistics Admitted Patient Care, NHS Digital
