# Supplementary material for: Numbers and types of neurological emergencies in England and the influence of socioeconomic deprivation: a retrospective analysis of hospital episode statistics data
Source: BMJ Open. 2022 Nov 4;12(11):e061843. doi: 10.1136/bmjopen-2022-061843 (PMC9639083; doi:10.1136/bmjopen-2022-061843)
Supplement: Author's manuscript [file bmjopen-2022-061843.draft_revisions.pdf]

BMJ Open

BMJ Open is committed to open peer review. As part of this commitment we make the peer review history of every article we publish publicly available.

When an article is published we post the peer reviewers' comments and the authors' responses online. We also post the versions of the paper that were used during peer review. These are the versions that the peer review comments apply to.

The versions of the paper that follow are the versions that were submitted during the peer review process. They are not the versions of record or the final published versions. They should not be cited or distributed as the published version of this manuscript.

BMJ Open is an open access journal and the full, final, typeset and author-corrected version of record of the manuscript is available on our site with no access controls, subscription charges or pay-per-view fees (http://bmjopen.bmj.com).

If you have any questions on BMJ Open's open peer review process please email info.bmjopen@bmj.com

BMJ Open

An analysis of the number and types of neurological emergencies in England and the influence of socio-economic deprivation

Journal:	*BMJ Open*
Manuscript ID	bmjopen-2022-061843
Article Type:	Original research
Date Submitted by the Author:	10-Feb-2022
Complete List of Authors:	Jackson, Michael; United Kingdom Department of Health and Social Care, Office for Health Improvement and Disparities (OHID)<br>Szczepaniak, Marta; United Kingdom Department of Health and Social Care, Office for Health Improvement and Disparities (OHID)<br>Wall, Jasmine; Lancaster University; Lancashire Teaching Hospitals NHS Foundation Trust<br>Maskery, Mark; Lancaster University; Lancashire Teaching Hospitals NHS Foundation Trust<br>Mummery, Catherine; University College London Hospitals NHS Foundation Trust<br>Morrish, Paul; Great Western Hospital Foundation NHS Trust<br>Williams, Adrian; University Hospitals Birmingham NHS Foundation Trust; NHS England<br>Knight, Joanne; Lancaster University<br>Emsley, Hedley; Lancaster University; Lancashire Teaching Hospitals NHS Foundation Trust
Keywords:	Epilepsy < NEUROLOGY, Neurology < INTERNAL MEDICINE, Public health < INFECTIOUS DISEASES

SCHOLARONE™
Manuscripts

I, the Submitting Author has the right to grant and does grant on behalf of all authors of the Work (as defined in the below author licence), an exclusive licence and/or a non-exclusive licence for contributions from authors who are: i) UK Crown employees; ii) where BMJ has agreed a CC-BY licence shall apply, and/or iii) in accordance with the terms applicable for US Federal Government officers or employees acting as part of their official duties; on a worldwide, perpetual, irrevocable, royalty-free basis to BMJ Publishing Group Ltd ("BMJ") its licensees and where the relevant Journal is co-owned by BMJ to the co-owners of the Journal, to publish the Work in this journal and any other BMJ products and to exploit all rights, as set out in our licence.

The Submitting Author accepts and understands that any supply made under these terms is made by BMJ to the Submitting Author unless you are acting as an employee on behalf of your employer or a postgraduate student of an affiliated institution which is paying any applicable article publishing charge ("APC") for Open Access articles. Where the Submitting Author wishes to make the Work available on an Open Access basis (and intends to pay the relevant APC), the terms of reuse of such Open Access shall be governed by a Creative Commons licence – details of these licences and which Creative Commons licence will apply to this Work are set out in our licence referred to above.

Other than as permitted in any relevant BMJ Author's Self Archiving Policies, I confirm this Work has not been accepted for publication elsewhere, is not being considered for publication elsewhere and does not duplicate material already published. I confirm all authors consent to publication of this Work and authorise the granting of this licence.

For peer review only

An analysis of the number and types of neurological emergencies in England and the influence of socio-economic deprivation

A retrospective empirical research study

Jackson M[1], Szczepaniak M[1], Wall J[2,3], Maskery M[2,3], Mummery C[4], Morrish P[5], Williams A[6,7], Knight J[1,2], Emsley HCA[1,2,3]

[1]Neurology & Dementia Intelligence team part of Public Health Analysis Group, Office for Health Improvement and Disparities (OHID), Department of Health and Social Care, UK (Formerly Public Health England); [2]Lancaster Medical School, Lancaster University, Lancaster, UK; [3]Lancashire Teaching Hospitals NHS Foundation Trust, Preston, UK; [4]Dementia Research Centre, National Hospital for Neurology and Neurosurgery, University College London Hospitals NHS Foundation Trust, London, UK; [5]Greater Western Hospitals NHS Foundation Trust, Swindon, UK; [6]University Hospitals Birmingham NHS Foundation Trust, Birmingham, UK; [7]Neuroscience Clinical Reference Group, NHS England, London, UK

Author email addresses

Michael Jackson	michael.jackson@dhsc.gov.uk
Marta Szczepaniak	marta.szczepaniak@dhsc.gov.uk
Jasmine Wall	jasmine.wall@lthtr.nhs.uk
Mark Maskery	mark.maskery@lthtr.nhs.uk
Cath Mummery	cath.mummery@nhs.net
Paul Morrish	p.morrish@nhs.net
Adrian Williams	adrian.williams@uhb.nhs.uk
Jo Knight	jo.knight@lancaster.ac.uk
Hedley Emsley*	hedley.emsley@lancaster.ac.uk

*corresponding author address. Department of Neurology, Royal Preston Hospital, Lancashire Teaching Hospitals NHS Foundation Trust, Sharoe Green Lane, Preston, PR2 9HT

For peer review only

Abstract

Objectives In this first large-scale analysis of neurological emergency admissions in England, we determine the number and types of emergency admissions with neurological emergency diagnostic codes, how many are under the care of a neurologist or neurosurgeon, and how such admissions vary by levels of deprivation

Design Retrospective empirical research employing a derived list of neurological emergency diagnostic codes

Setting This study used the Hospital Episode Statistics dataset for the financial year 2019/2020 based on 17 million in-year inpatient admissions in England including 6.5 million (100%) emergency admissions with any diagnosis codes

Results There were 1.4 million (21.2%) emergency inpatient admissions with a mention of any neurological code, approx. 248,455 (3.8%) with mention of a specific neurological emergency code from the derived list, and 72,485 (1.1%) included such a code as the primary reason for admission. The highest number of in-year admissions for adults was for epilepsy (145,995), with epilepsy as the primary diagnostic code in 10.9% of these. Acute nerve root/spinal cord syndrome (41,215), head injury (29,235) and subarachnoid haemorrhage (18,505) accounted for the next three highest number of admissions. 1.4% of in-year emergency hospital admissions with mention of a neurological emergency code were under the care of a neurologist or neurosurgeon, with only 0.9% of admissions with mention of an epilepsy code under a neurologist. The number of admissions varied significantly by social deprivation, with this pattern more apparent for epilepsy and most evident for functional neurological disorders, where the greatest number were in the most deprived and the fewest in the least deprived.

Conclusions This study has identified important findings in relation to the burden of neurological emergency admissions but further work is needed, with greater clinical engagement in diagnostic coding, to better understand the implications for workforce and changes to service delivery needing to be implemented.

For peer review only

Introduction

Medical emergency admissions continue to rise across the UK. They are more than twice as likely among the most deprived population decile by comparison with mean deprivation, reinforcing the point that challenges are social as well as medical[1,2]. Many emergency admissions to hospital are considered to be avoidable and patients often stay in hospital longer than is necessary[3]. Emergency admissions are also disruptive of elective care delivery, prolonging waiting times. Hospital Episodes Statistics (HES) data for 2016/17 suggest that approximately 18% of medical emergency admissions included a mention of neurological conditions including stroke and dementia, totalling 1.09 million, a rise of 21% over the previous 5 years[4]. Excess mortality has also been reported among patients with epilepsy[5]. At a time when costs are rising and resources are increasingly under pressure, it is all the more important to have a better understanding of neurological emergency admissions and underlying factors such as social deprivation.

There has been little by way of systematic evaluation of data pertaining to neurological emergency admissions. Previous work has used a much broader definition, counting neurological conditions present in patients admitted as an emergency, including stroke and dementia, rather than neurological emergencies *per se*. The present work has by necessity involved the creation of a list defining neurological emergencies. Much remains unknown about neurological emergency admissions, including their total number, the distribution of neurological conditions, and the effect of socio-economic deprivation. There have been some studies investigating the influence of comorbidities and race in the United States [6,7]. This study represents the first large scale analysis of neurological emergency admissions in England, and the first OHID-academic partnership in neurology. For the first time, the data provide some insight into the number and types of emergency admissions with neurological emergency codes, the number of such admissions under the care of a neurologist, and how such admissions vary on the basis of factors such as levels of deprivation.

For peer review only

Methods

Defining neurological emergencies

For the purposes of this work it was necessary to create a list of conditions typically regarded as neurological emergencies, and to map these to the relevant ICD-10 codes. An initial set of neurological conditions was proposed by AW as the basis for the study, comprising causes of acute paralysis such as Guillain-Barré syndrome, myasthenic crisis, encephalitis, spinal cord compression and status epilepticus. The ICD-10 codes most closely matching these 'index' entities were compiled into a list. Other ICD-10 codes in close hierarchical proximity to the 'index' ICD-10 codes were added from a previously published Public Health England (PHE) neurology code collection [8], which comprised 581 4-digit ICD-10 codes for 19 condition groups. The 'long list' of candidate neurological emergency codes, consisting of 136 ICD-10 codes, was reviewed iteratively by the project team and clinicians working alongside, and refined into a short list of 96 4-digit ICD-10 codes (appendix 1) for use in the work reported here, subsequently referred to as the 'derived list' [of neurological emergency codes]. The selection process used clinical judgement to decide which conditions or groups of conditions to include or exclude. Of particular note, stroke, despite being a classical neurological emergency, was excluded from this exercise given that stroke is already the subject of the existing national audit system (SSNAP). Codes for conditions presenting with secondary headache have been included; codes for primary headache disorders have not been included in the present work. Also of note was the decision to include functional neurological disorders (FNDs) on the basis that we believed these to contribute significantly to the total number of emergency hospital admissions for neurological conditions, as well as the need for patients with FND to be referred to specialists with expertise in neurological diagnosis [9]. FNDs is a term given to neurological features that are not due to any physical neurological disorder. We have defined relevant terminology in Box 1.

Defining study group

The hospital activity included in this study were admissions to hospital occurring during the financial year 2019/2020 in England – described as an in-year admission, where the admission was classed as an emergency (non-elective) admission to hospital and where the selected neurological emergency codes are in any diagnosis position, on any consultant episode during the hospital stay.

Data source, data extraction and descriptive statistics

The data source used in this study is the Admitted Patient Care version of the Hospital Episode Statistics dataset for the financial year 2019/2020. Data extraction was undertaken by the OHID Neurology Dementia Intelligence team using Structured Query Language (SQL) with further data manipulation, including descriptive statistics, using Microsoft Excel. Counts and percentages were extracted and/or calculated for various categories, confidence intervals were estimated and data was visualised in a range of chart types.

Patient and public involvement

This was a retrospective empirical research study based on a secondary analysis of routinely collected anonymised clinical data. Patient and public involvement was not applicable.

For peer review only

Ethics statement

Ethics Committee approval was not required as the research presented in this manuscript comprises a secondary analysis of routinely collected anonymised clinical data.

Box 1. Relevant terminology

People admitted to hospital will always have a responsible medical officer (consultant) assigned for the time under that consultant, or episode (**consultant episode**).

The consultant episode at the start of the hospital stay is the **admission episode**; the episode at the end of the hospital spell is called the **discharge episode**.

Hospital admissions are the start of **hospital spells**, which comprise one or more consultant episodes.

ICD-10 – International Statistical Classification of Diseases and Related Health Problems (ICD) (10th edition) codes, or **hospital diagnosis codes**. ICD-10 is a legally mandated health data standard.

Conditions present during the consultant episode can be described using up to 20 corresponding ICD-10 codes . Hospital stays with multiple consultant episodes can have very differing collections of ICD-10 codes recorded on each episode.

The ICD-10 code that is recorded in the first position on a consultant episode is known as the **primary diagnosis**. Subsequent codes on the same episodes are considered **secondary diagnoses** and may represent conditions of lesser importance for this particular consultant episode or other long-term conditions/co-morbidities of the patient.

Hospital episode statistics (HES) are financial year based. Stays in hospital can cross the financial year timelines, therefore the number of admissions and discharges in any one financial year may not be equal. **In-year hospital admissions** relates to the count of all hospital stays that start in the financial year. **In-year hospital discharges** relate to the count of all hospital stays that end during the financial year. **HES ID** is a pseudonymised unique identifier for each individual patient, which can be used to group hospital episodes and spells by individual

Results

Emergency inpatient admissions with any neurological codes or neurological emergency codes

Table 1 shows totals for inpatient activity for all ages by admission type for 2019/2020. Of a total of over 17 million inpatient admissions, 6.5 million were emergency admissions. Among these, 1.4 million (21%) were emergency inpatient admissions with a mention of any neurological code (including dementia and stroke). Emergency inpatient admissions with a specific neurological emergency code from the derived list (appendix 1) represented 3.8% of all emergency inpatient admissions, and such a code was the primary reason for admission in 1.1% of all emergency inpatient admissions. Among all emergency adult in-year admissions by spell with specific neurological diagnosis codes from the derived list 132,485 (54.4%) comprised a single episode.

The number of unique adult patients (using HESID as a proxy) with in-year admissions by spell with specific neurological diagnosis codes from the derived list was 167,735. 75.6% (126,800) of these individuals have only one admission.

Table 1: Inpatient hospital activity by admission type; all ages; England; 2019/20

	Counts	Percentage of all emergency inpatient admissions
All inpatient admissions: in-year admissions by spell with any diagnosis codes	17,183,220	NA
Emergency inpatient admissions: in-year admissions by spell with any diagnosis codes	6,506,970	100%
Emergency inpatient admissions with a mention of any neurological code (including dementia and stroke): in-year admissions by spell	1,380,975	21.2%
Emergency inpatient admissions with neurological emergency codes: in-year admissions by spell with specific neurological diagnosis codes	248,455	3.8%
Emergency inpatient admissions with neurological emergency code as primary reason for admission: in-year admissions by spell with specific neurological diagnosis codes	72,485	1.1%

Source: OHID Neurology Dementia Intelligence using Hospital Episode Statistics Admited Patient Care, NHS Digital

For peer review only

Neurological conditions

Table 2 shows adult emergency inpatient hospital admissions with a mention of neurological emergency codes. Among a total of 237,755 such admissions, 65,060 (27.4%) had a neurological emergency code as a primary diagnostic code on the starting episode. Similar percentages were noted for acute paralysis (24.5%), functional disorders (29.8%), acute nerve root/cord syndrome (30.7%) and encephalitis (34.2%).

However we see a strikingly different pattern for epilepsy, subarachnoid haemorrhage (SAH) and head injury (HI). The highest number of in-year admissions was for epilepsy (145,995), yet the percentage of those with epilepsy as the primary diagnostic code was only 10.9%. By comparison, the primary diagnostic codes for head injury appeared in 73.1% of 29,235 admissions for this condition, and similarly for subarachnoid haemorrhage the primary diagnostic code appeared in 57.9% of 18,505 admissions.

Table 2: Emergency inpatient hospital admissions with a mention of neurological emergency codes; adults aged 18 years and over; England; 2019/20

	In-year admissions	In-year admissions with primary diagnosis on starting episode	Primary diagnosis as % of in-year admissions
neurological emergencies	237,755	65,060	27.4%
emergency acute nerve root/cord syndrome	41,215	12,655	30.7%
emergency acute paralysis	2,600	635	24.5%
emergency encephalitis	5,240	1,790	34.2%
emergency epilepsy	145,995	15,945	10.9%
emergency functional disorders	6,545	1,950	29.8%
emergency head injuries	29,235	21,365	73.1%
emergency subarachnoid haemorrhage	18,505	10,720	57.9%

Source: OHID Neurology Dementia Intelligence using Hospital Episode Statistics Admited Patient Care, NHS Digital

For peer review only

Proportion of admissions under a neurologist

Figure 1 shows the percentage of in-year adult emergency hospital admissions with mention of a neurological emergency code under the care of a consultant in neurology or neurosurgery. Overall, among 237,755 such admissions, 3,230 (1.4%) were under the care of a consultant neurologist and 10,760 (4.5%) were under the care of a consultant neurosurgeon, with the majority of the admissions under a neurosurgeon having SAH or HI diagnostic codes (see supplementary table 1). Only 1,315 (0.9%) of admissions with mention of an epilepsy diagnostic code were under the care of a consultant neurologist.

Figure 1. Percentage of in-year emergency hospital admissions with mention of neurological emergency codes admitted under the care of a consultant neurologist or neurosurgeon; adults aged 18 years and over; England; 2019/20; source: OHID Neurology Dementia Intelligence using Hospital Episode Statistics Admitted Patient Care, NHS Digital.

[see separate figure 1 file]

For peer review only

Influence of socio-economic deprivation

Figure 2 shows a proxy for the number of people admitted (total, functional, epilepsy, SAH) by deciles of deprivation using the Indices of Multiple Deprivation [10]. This shows that the number of people admitted varies significantly by social deprivation (see also supplementary table 2). This pattern is seen for the total admissions with mention of neurological emergency codes, but is more apparent for epilepsy and is most striking for functional neurological disorders, where the greatest number were in the most deprived and the fewest in the least deprived. By comparison, admissions with mention of SAH did not exhibit any apparent variation by social deprivation.

Figure 2. Proportion of unique HESIDs (proxy for individuals) with an in-year neurological emergency hospital admission, by decile of deprivation and condition group; adults aged 18 years and over; Indices of Multiple Deprivation 2019; England; 2019/20; source: OHID Neurology Dementia Intelligence using Hospital Episode Statistics Admitted Patient Care, NHS Digital.

[see separate figure 2 file]

For peer review only

Discussion

In this first large-scale analysis of 1.4 million emergency inpatient admissions in England for 2019/2020 with a mention of any neurological code, there were 248,455 with mention of a specific neurological emergency code from the derived list. The highest number was for epilepsy (145,995) with the next three most frequent causes being acute nerve root/spinal cord syndrome, head injury and subarachnoid haemorrhage. 1.4% of admissions with mention of a neurological emergency code were under a neurologist or neurosurgeon, with only 0.9% of those with mention of an epilepsy code under a neurologist. The greatest number of admissions were in the most deprived, particularly for epilepsy and functional neurological disorders. Being able to determine the proportion of emergency admissions that constitute neurological emergency presentations is clearly crucial for service and clinical pathway design and workforce planning.

It was necessary to formulate a list of neurological emergencies and associated list of ICD-10 codes as a prelude to the analyses in this study. We took a pragmatic approach based on current UK practice, taking into account factors such as existing well defined pathways of care (eg acute stroke) or the absence of well defined pathways (eg FND). Although perhaps not classically considered neurological emergencies, FND presentations are increasingly common. This is with respect to their impact both the individual and upon healthcare resources, resources which are often used inappropriately and not in alignment with the needs of such patients.

We found that among adult emergency admissions with mention of a neurological emergency code, this was the primary diagnostic code in 27.4% overall. However, despite epilepsy accounting for the greatest number of admissions in our dataset, epilepsy appeared as the primary diagnostic code in only 10.9% by comparison with 73.1% for head injury 57.9% for SAH. It is important to note that epilepsy codes have been included among the neurological emergency codes but may reflect epilepsy as a comorbidity and not the reason for emergency admission, hence the comparatively low proportion of patients with epilepsy codes as the primary diagnostic code. A proportion of patients assigned epilepsy codes may have had dissociative seizures (DS). Admissions with mention of epilepsy codes merit further analysis in future work, particularly given recent data on epilepsy related mortality [5]. The current analysis cannot explain the differences between conditions in the proportion of relevant primary diagnostic codes being assigned. However, it is conceivable that the high proportion of head injury admissions with head injury as the primary diagnostic code, and similarly for SAH, may reflect the readily apparent the relative ease and pace of establishing the primary reason for admission.

We found that among emergency admissions with mention of a neurological emergency code, 1.4% were under the care of a consultant neurologist and 4.5% were under the care of a consultant neurosurgeon; only 0.9% with mention of an epilepsy diagnostic code were under the care of a consultant neurologist. Many admissions with single episodes not under neurology or neurosurgery also suggests many patients may not receive input from these disciplines despite having neurological codes assigned. Although the percentage of admissions under a consultant neurologist may appear disproportionately low given the greater number of neurologists by comparison with neurosurgeons, this may partly reflect the different balance of commitments between outpatient and inpatient pathways between neurology and neurosurgery. These findings are in keeping with previous data suggesting that a low proportion of epilepsy emergency admissions are under a neurologist. Further work, such as the prospective capture of neurological emergency codes with greater clinical engagement to improve their accuracy, is needed, but our findings are in general agreement with the recognised urgent need to address workforce capacity to support neurological emergency admissions.

For peer review only

The present study highlights additional concerns, particularly the apparent relationship between admissions for FND and socioeconomic deprivation. We know that in many areas in England there is a significant gap in terms of provision of appropriate services for patients with FND [11], very likely to increase the likelihood of re-presentation as an emergency. A better understanding of emergency admissions for FND is needed to determine how clinical pathways can be improved for such patients. The retrospective nature of the present study limits interpretation of findings such as acute nerve root/spinal cord syndrome being arguably more frequent than expected; one possibility is a rise in 'scan-negative' cauda equina syndrome, itself another potential FND[9].

An important limitation of the methodology employed by this study is the inclusion of emergency admissions which mention neurological conditions, ie admissions which include codes deemed, as part of this study, relevant for neurological emergencies. These codes could appear at any position in the patient's diagnostic coding, and during any episode(s) comprising the given spell; implicit in this approach is the possibility that the neurological condition was not the reason for the emergency admission. This, of course, is different to the inclusion of neurological emergencies *per se*, where the primary cause of the emergency admission was neurological. Previous work has raised concern about the accuracy of current data collection methods, including the erroneous assignment of neurological codes [12].

Conclusion We have for the first time quantified the number of neurological emergency admissions across England, the distribution of conditions, the proportion under the care of a neurologist or neurosurgeon and evidence of variation by social deprivation. Further work, including improved clinical engagement with coding to ensure data accuracy, is required to understand the drivers of these findings. Further analyses are also needed to investigate issues such as the effect of hospital setting (eg district general hospital vs neurosciences centre), the point in the spell at which the neurological diagnosis is made and the influence of neurologist involvement. These activities will inform clinical pathway change across primary and secondary care to reduce avoidable admissions and improve care.

For peer review only

Contributorship statement

MJ, MS, JK and HE conceived and designed the work and jointly undertook the analysis and interpretation of the data. MJ, JK and HE jointly drafted the manuscript and revised it critically for important intellectual content. AW contributed to the conception of the work. JW, MM, CM, PM and AW contributed to the development of the derived list of neurological emergency diagnostic codes. All authors approved the final version.

The corresponding author attests that all listed authors meet authorship criteria and that no others meeting the criteria have been omitted.

Competing interests

The authors have no relevant competing interests to declare.

Funding

This study received no specific funding.

Data sharing statement

Information relating to access to HES data used for this study can be found at https://digital.nhs.uk/data-and-information/data-tools-and-services/data-services/hospital-episode-statistics/users-uses-and-access-to-hospital-episode-statistics.

For peer review only

References

1. McCormick B, Hill P-S, Poteliakhoff E. 2012. Are hospital services used differently in deprived areas? Evidence to identify commissioning challenges. http://chseo.org.uk/downloads/wp2-hospitalservices-deprivedareas.pdf (accessed December 2021)
2. Purdy S. 2010. Avoiding hospital admissions. London: The King's Fund. https://www.kingsfund.org.uk/sites/default/files/Avoiding-Hospital-Admissions-Sarah-Purdy-December2010.pdf (accessed January 2022)
3. National Audit Office. 2013. Emergency admissions to hospital: managing the demand. https://www.nao.org.uk/report/emergency-admissions-hospitals-managing-demand/ (accessed December 2021)
4. The Neurological Alliance. 2019. Neuro Numbers 2019. https://www.neural.org.uk/assets/pdfs/neuro-numbers-2019.pdf (accessed December 2021)
5. Public Health England. 2018. Deaths associated with neurological conditions data briefing. https://www.gov.uk/government/publications/deaths-associated-with-neurological-conditions (accessed January 2022)
6. Sajatovic M, Welter E, Tatsuoka C, Perzynski AT, Einstadter D. Electronic medical record analysis of emergency room visits and hospitalizations in individuals with epilepsy and mental illness comorbidity. Epilepsy Behav. 2015 Sep;50:55-60. doi: 10.1016/j.yebeh.2015.05.016. Epub 2015 Jun 25. PMID: 26119621; PMCID: PMC4793963.
7. Fantaneanu TA, Hurwitz S, van Meurs K, Llewellyn N, O'Laughlin KN, Dworetzky BA. Racial differences in Emergency Department visits for seizures. Seizure. 2016 Aug;40:52-6. doi: 10.1016/j.seizure.2016.06.007. Epub 2016 Jun 16. PMID: 27344498.
8. Public Health England. 2021. Neurology and Dementia Intelligence: ICD10 code collections. https://fingertips.phe.org.uk/profile-group/mental-health/supporting-information/ndi-code-collections (accessed December 2021)
9. Bennett K, Diamond C, Hoeritzauer I, Gardiner P, McWhirter L, Carson A, Stone J. A practical review of functional neurological disorder (FND) for the general physician. Clin Med (Lond). 2021 Jan;21(1):28-36. doi: 10.7861/clinmed.2020-0987. PMID: 33479065; PMCID: PMC7850207.
10. Ministry of Housing, Communities & Local Government. 2019. English indices of deprivation 2019. https://www.gov.uk/government/statistics/english-indices-of-deprivation-2019 (accessed December 2021)
11. O'Keeffe S, Chowdhury I, Sinanaj A, Ewang I, Blain C, Teodoro T, Edwards M, Yogarajah M. A Service Evaluation of the Experiences of Patients With Functional Neurological Disorders Within the NHS. Front Neurol. 2021 May 31;12:656466. doi: 10.3389/fneur.2021.656466. PMID: 34135848; PMCID: PMC8200476.
12. Kanagaratnam M, Boodhoo A, MacDonald BK, Nitkunan A. Prevalence of acute neurology: a 2-week snapshot in a district general hospital. Clin Med (Lond). 2020;20(2):169-173. doi:10.7861/clinmed.2019-0377

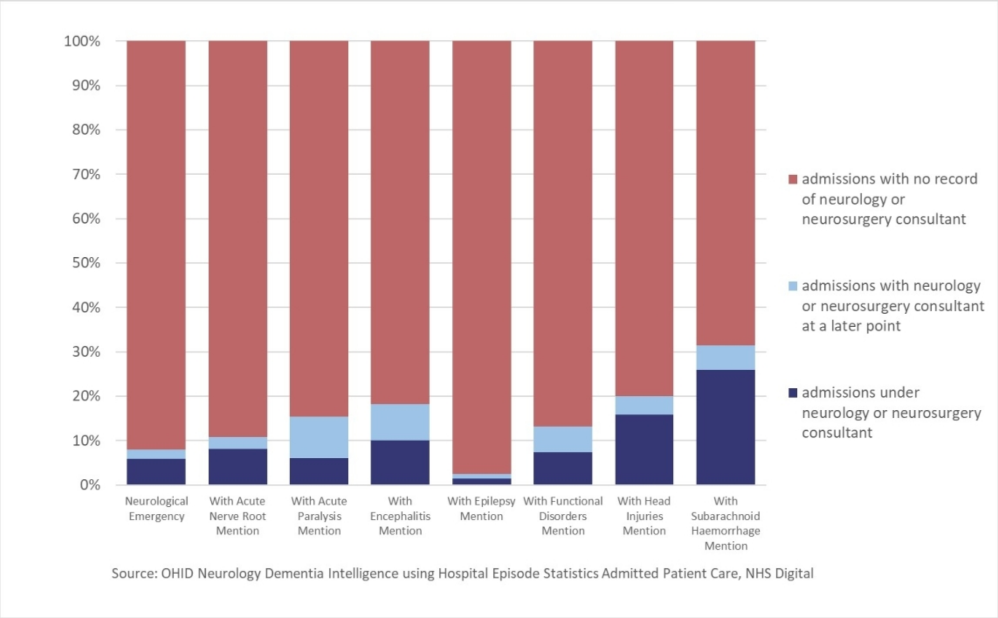

Figure 1. Percentage of in-year emergency hospital admissions with mention of neurological emergency codes admitted under the care of a consultant neurologist or neurosurgeon; adults aged 18 years and over; England; 2019/20; source: OHID Neurology Dementia Intelligence using Hospital Episode Statistics Admitted Patient Care, NHS Digital.

256x159mm (150 x 150 DPI)

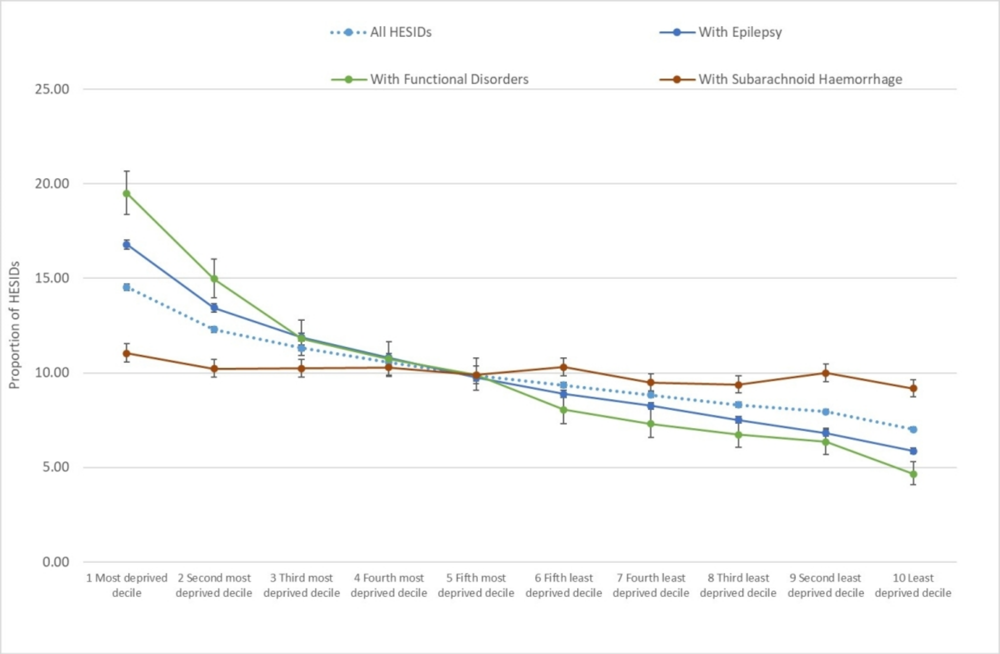

Figure 2. Proportion of unique HESIDs (proxy for individuals) with an in-year neurological emergency hospital admission, by decile of deprivation and condition group; adults aged 18 years and over; Indices of Multiple Deprivation 2019; England; 2019/20; source: OHID Neurology Dementia Intelligence using Hospital Episode Statistics Admitted Patient Care, NHS Digital.

258x168mm (150 x 150 DPI)

Supplementary Table 1: Percentage of in-year emergency hospital admissions with mention of neurological emergency codes admitted under the care of a consultant neurologist or neurosurgeon; adults aged 18 years and over; England; 2019/20

	Neurological Emergency	With Acute Nerve Root Mention	With Acute Paralysis Mention	With Encephalitis Mention	With Epilepsy Mention	With Functional Disorders Mention	With Head Injuries Mention	With Subarachnoid Haemorrhage Mention
admissions under neurology consultant	3,230	545	145	270	1,315	445	305	440
%	*1.36%*	*1.32%*	*5.58%*	*5.19%*	*0.90%*	*6.80%*	*1.04%*	*2.38%*
admissions under neurosurgery consultant	10,760	2,905	10	255	810	40	4,360	4,375
%	*4.52%*	*7.05%*	*0.42%*	*4.89%*	*0.56%*	*0.63%*	*14.91%*	*23.63%*
admissions under neurology or neurosurgery consultant	13,860	3,355	155	525	2,120	485	4,655	4,800
%	*5.83%*	*8.15%*	*6.00%*	*10.06%*	*1.45%*	*7.40%*	*15.92%*	*25.95%*
admissions with neurology consultant at a later point	3,150	435	250	410	1,355	370	390	320

For peer review only

%	*1.32%*	*1.06%*	*9.58%*	*7.81%*	*0.93%*	*5.67%*	*1.33%*	*1.72%*
admissions with neurosurgery consultant at a later point	2,265	795	*	55	165	10	945	835
%	*0.95%*	*1.93%*	*0.00%*	*1.07%*	*0.11%*	*0.17%*	*3.23%*	*4.52%*
admissions with neurology or neurosurgery consultant at a later point	5,000	1,110	245	430	1,465	375	1,200	1,010
%	*2.10%*	*2.70%*	*9.35%*	*8.21%*	*1.00%*	*5.72%*	*4.11%*	*5.49%*
admissions with no record of neurology or neurosurgery consultant	218,890	36,745	2,200	4,280	142,410	5,690	23,380	12,690
%	*92.07%*	*89.15%*	*84.64%*	*81.73%*	*97.54%*	*86.89%*	*79.97%*	*68.56%*
**in year admissions**	**237,755**	**41,215**	**2,600**	**5,240**	**145,995**	**6,545**	**29,235**	**18,505**
Source: OHID Neurology Dementia Intelligence using Hospital Episode Statistics Admited Patient Care, NHS Digital								

For peer review only

Supplementary Table 2

**Deciles of Deprivation England**		**All HESIDs**	**With Epilepsy**	**With Functional Disorders**	**With Subarachnoid Haemorrhage**
**1 Most deprived**	Count	23,600	14,970	910	1,770
	Percentage	14.5%	16.8%	19.5%	11.0%
**2**	Count	19,965	11,990	700	1,640
	Percentage	12.3%	13.4%	15.0%	10.2%
**3**	Count	18,365	10,595	555	1,640
	Percentage	11.3%	11.9%	11.8%	10.2%
**4**	Count	17,175	9,640	500	1,650
	Percentage	10.6%	10.8%	10.7%	10.3%
**5**	Count	16,005	8,700	465	1,590
	Percentage	9.9%	9.8%	9.9%	9.9%
**6**	Count	15,190	7,920	375	1,655
	Percentage	9.4%	8.9%	8.1%	10.3%
**7**	Count	14,340	7,365	340	1,525
	Percentage	8.8%	8.3%	7.3%	9.5%
**8**	Count	13,510	6,700	315	1,505
	Percentage	8.3%	7.5%	6.7%	9.4%
**9**	Count	12,920	6,080	300	1,605
	Percentage	8.0%	6.8%	6.3%	10.0%
**10 Least deprived**	Count	11,375	5,225	220	1,470
	Percentage	7.0%	5.9%	4.7%	9.2%

Source: OHID Neurology Dementia Intelligence using Hospital Episode Statistics Admitted Patient Care, NHS Digital

For peer review only

Appendix 1

Neurological Emergency ICD10 codes – 96 codes (short list)

**NeuroEmergencyConditionGroup**	**NeuroEmergencyICD10CodeHES**	**ICD10_Level3Header**
Acute paralysis	A521	Symptomatic neurosyphilis
Encephalitis	A830	Japanese encephalitis
Encephalitis	A831	Western equine encephalitis
Encephalitis	A832	Eastern equine encephalitis
Encephalitis	A833	St Louis encephalitis
Encephalitis	A834	Australian encephalitis
Encephalitis	A835	California encephalitis
Encephalitis	A836	Rocio virus disease
Encephalitis	A838	Other mosquito-borne viral encephalitis
Encephalitis	A839	Mosquito-borne viral encephalitis
Encephalitis	A840	Far Eastern tick-borne encephalitis [Russian spring-summer encephalitis]
Encephalitis	A841	Central European tick-borne encephalitis
Encephalitis	A848	Other tick-borne viral encephalitis
Encephalitis	A849	Tick-borne viral encephalitis
Encephalitis	A850	Enteroviral encephalitis
Encephalitis	A851	Adenoviral encephalitis
Encephalitis	A852	Arthropod-borne viral encephalitis, unspecified
Encephalitis	A858	Other specified viral encephalitis

For peer review only

Encephalitis	A86X	Unspecified viral encephalitis
Functional Disorders	F440	Dissociative amnesia
Functional Disorders	F441	Dissociative fugue
Functional Disorders	F442	Dissociative stupor
Functional Disorders	F443	Trance and possession disorders
Functional Disorders	F444	Dissociative motor disorders
Functional Disorders	F445	Dissociative convulsions
Functional Disorders	F446	Dissociative anaesthesia and sensory loss
Functional Disorders	F447	Mixed dissociative conversion disorders
Functional Disorders	F448	Other dissociative conversion disorders
Functional Disorders	F449	Dissociative conversion disorder unspecified
Encephalitis	G040	Acute disseminated encephalitis
Encephalitis	G041	Tropical spastic paraplegia
Encephalitis	G042	Bacterial meningoencephalitis and meningomyelitis NEC
Encephalitis	G048	Other encephalitis myelitis and encephalomyelitis
Encephalitis	G049	Encephalitis myelitis and encephalomyelitis unspecified
Encephalitis	G050	Encephalitis myelitis and encephalomyelitis in bacterial diseases classified elsewhere
Encephalitis	G051	Encephalitis myelitis and encephalomyelitis in viral diseases classified elsewhere
Encephalitis	G052	Encephalitis myelitis and encephalomyelitis in other infectious and parasitic diseases classified elsewhere
Encephalitis	G058	Encephalitis myelitis and encephalomyelitis in other diseases classified elsewhere
Acute nerve root/cord syndrome	G114	Hereditary spastic paraplegia
Epilepsy	G409	Epilepsy unspecified
Epilepsy	G410	Grand mal status epilepticus
Epilepsy	G411	Petit mal status epilepticus

For peer review only

Epilepsy	G412	Complex partial status epilepticus
Epilepsy	G418	Other status epilepticus
Epilepsy	G419	Status epilepticus unspecified
Acute nerve root/cord syndrome	G549	Nerve root and plexus disorder
Acute nerve root/cord syndrome	G551	Nerve root and plexus compressions in intervertebral disc disorders
Acute paralysis	G610	Guillain-Barre syndrome
Acute nerve root/cord syndrome	G820	Flaccid paraplegia
Acute nerve root/cord syndrome	G821	Spastic paraplegia
Acute nerve root/cord syndrome	G822	Paraplegia unspecified
Acute nerve root/cord syndrome	G823	Flaccid tetraplegia
Acute nerve root/cord syndrome	G824	Spastic tetraplegia
Acute nerve root/cord syndrome	G825	Tetraplegia unspecified
Acute nerve root/cord syndrome	G952	Cord compression unspecified
Acute nerve root/cord syndrome	G992	Myelopathy in diseases classified elsewhere
Subarachnoid haemorrhage	I600	Subarachnoid haemorrhage from carotid siphon and bifurcation
Subarachnoid haemorrhage	I601	Subarachnoid haemorrhage from middle cerebral artery

For peer review only

Subarachnoid haemorrhage	I602	Subarachnoid haemorrhage from anterior communicating artery
Subarachnoid haemorrhage	I603	Subarachnoid haemorrhage from posterior communicating artery
Subarachnoid haemorrhage	I604	Subarachnoid haemorrhage from basilar artery
Subarachnoid haemorrhage	I605	Subarachnoid haemorrhage from vertebral artery
Subarachnoid haemorrhage	I606	Subarachnoid haemorrhage from other intracranial arteries
Subarachnoid haemorrhage	I607	Subarachnoid haemorrhage from intracranial artery, unspecified
Subarachnoid haemorrhage	I608	Other subarachnoid haemorrhage
Subarachnoid haemorrhage	I609	Subarachnoid haemorrhage, unspecified
Subarachnoid haemorrhage	I690	Sequelae of subarachnoid haemorrhage
Acute nerve root/cord syndrome	M470	Anterior spinal and vertebral artery compression syndromes
Acute nerve root/cord syndrome	M471	Other spondylosis with myelopathy
Acute nerve root/cord syndrome	M490	Tuberculosis of spine
Acute nerve root/cord syndrome	M500	Cervical disc disorder with myelopathy - linked to G992A
Acute nerve root/cord syndrome	M501	Cervical disc disorder with radiculopathy
Acute nerve root/cord syndrome	M510	Lumbar and other intervertebral disc disorders with myelopathy
Acute nerve root/cord syndrome	M511	M51.1(dagger) Lumbar and other intervertebral disc disorders with radiculopathy (G55.1(asterix))
Acute nerve root/cord syndrome	M612	Paralytic calcification and ossification of muscle (Myositis ossificans associated with quadriplegia or paraplegia)
Head Injuries	S040	Injury of optic nerve and pathways

For peer review only

Head Injuries	S041	Injury of oculomotor nerve
Head Injuries	S042	Injury of trochlear nerve
Head Injuries	S043	Injury of trigeminal nerve
Head Injuries	S044	Injury of abducent nerve
Head Injuries	S045	Injury of facial nerve
Head Injuries	S046	Injury of acoustic nerve
Head Injuries	S047	Injury of accessory nerve
Head Injuries	S048	Injury of other cranial nerves
Head Injuries	S049	Injury of unspecified cranial nerve
Head Injuries	S060	Concussion
Head Injuries	S061	Traumatic cerebral oedema
Head Injuries	S062	Diffuse brain injury
Head Injuries	S063	Focal brain injury
Head Injuries	S064	Epidural haemorrhage
Head Injuries	S065	Traumatic subdural haemorrhage
Subarachnoid haemorrhage	S066	Traumatic subarachnoid haemorrhage
Head Injuries	S067	Intracranial injury with prolonged coma
Head Injuries	S068	Other intracranial injuries
Head Injuries	S069	Intracranial injury unspecified
Acute nerve root/cord syndrome	T093	Injury of spinal cord level unspecified

For peer review only

The RECORD statement – checklist of items, extended from the STROBE statement, that should be reported in observational studies using routinely collected health data.

	Item No.	STROBE items	Location in manuscript where items are reported	RECORD items	Location in manuscript where items are reported
**Title and abstract**					
	1	(a) Indicate the study's design with a commonly used term in the title or the abstract (b) Provide in the abstract an informative and balanced summary of what was done and what was found	Abstract, p2	RECORD 1.1: The type of data used should be specified in the title or abstract. When possible, the name of the databases used should be included.<br><br>RECORD 1.2: If applicable, the geographic region and timeframe within which the study took place should be reported in the title or abstract.<br><br>RECORD 1.3: If linkage between databases was conducted for the study, this should be clearly stated in the title or abstract.	Abstract, p2<br><br>Abstract, p2<br><br>Not applicable
**Introduction**					
Background rationale	2	Explain the scientific background and rationale for the investigation being reported	Introduction, p3		
Objectives	3	State specific objectives, including any prespecified hypotheses	Introduction, p3		
**Methods**					
Study Design	4	Present key elements of study design early in the paper	Methods, pp4-5		
Setting	5	Describe the setting, locations, and relevant dates, including periods of recruitment, exposure, follow-up, and data collection	Methods, pp4-5		

For peer review only

Participants	6	*(a) Cohort study* - Give the eligibility criteria, and the sources and methods of selection of participants. Describe methods of follow-up<br>*Case-control study* - Give the eligibility criteria, and the sources and methods of case ascertainment and control selection. Give the rationale for the choice of cases and controls<br>*Cross-sectional study* - Give the eligibility criteria, and the sources and methods of selection of participants<br><br>*(b) Cohort study* - For matched studies, give matching criteria and number of exposed and unexposed<br>*Case-control study* - For matched studies, give matching criteria and the number of controls per case	Retrospective empirical research based on routine healthcare data	RECORD 6.1: The methods of study population selection (such as codes or algorithms used to identify subjects) should be listed in detail. If this is not possible, an explanation should be provided.<br><br>RECORD 6.2: Any validation studies of the codes or algorithms used to select the population should be referenced. If validation was conducted for this study and not published elsewhere, detailed methods and results should be provided.<br><br>RECORD 6.3: If the study involved linkage of databases, consider use of a flow diagram or other graphical display to demonstrate the data linkage process, including the number of individuals with linked data at each stage.	Methods, pp4-5<br><br>Methods, pp4-5<br><br>Not applicable
Variables	7	Clearly define all outcomes, exposures, predictors, potential confounders, and effect modifiers. Give diagnostic criteria, if applicable.	Methods, pp4-5 and appendix 1	RECORD 7.1: A complete list of codes and algorithms used to classify exposures, outcomes, confounders, and effect modifiers should be provided. If these cannot be reported, an explanation should be provided.	Appendix 1
Data sources/ measurement	8	For each variable of interest, give sources of data and details of methods of assessment (measurement).<br>Describe comparability of assessment methods if there is more than one group	Methods, pp4-5		

For peer review only

Bias	9	Describe any efforts to address potential sources of bias	Methods, pp4-5		
Study size	10	Explain how the study size was arrived at	Methods, pp4-5		
Quantitative variables	11	Explain how quantitative variables were handled in the analyses. If applicable, describe which groupings were chosen, and why	Methods, pp4-5		
Statistical methods	12	(a) Describe all statistical methods, including those used to control for confounding<br>(b) Describe any methods used to examine subgroups and interactions<br>(c) Explain how missing data were addressed<br>(d) *Cohort study* - If applicable, explain how loss to follow-up was addressed<br>*Case-control study* - If applicable, explain how matching of cases and controls was addressed<br>*Cross-sectional study* - If applicable, describe analytical methods taking account of sampling strategy<br>(e) Describe any sensitivity analyses	Methods, pp4-5		
Data access and cleaning methods		..		RECORD 12.1: Authors should describe the extent to which the investigators had access to the database population used to create the study population.	Methods, pp4-5<br>Methods, pp4-5

For peer review only

				RECORD 12.2: Authors should provide information on the data cleaning methods used in the study.	
Linkage		..		RECORD 12.3: State whether the study included person-level, institutional-level, or other data linkage across two or more databases. The methods of linkage and methods of linkage quality evaluation should be provided.	Not applicable
**Results**					
Participants	13	(a) Report the numbers of individuals at each stage of the study (*e.g.*, numbers potentially eligible, examined for eligibility, confirmed eligible, included in the study, completing follow-up, and analysed)<br>(b) Give reasons for non-participation at each stage.<br>(c) Consider use of a flow diagram	Results, pp6-9	RECORD 13.1: Describe in detail the selection of the persons included in the study (*i.e.,* study population selection) including filtering based on data quality, data availability and linkage. The selection of included persons can be described in the text and/or by means of the study flow diagram.	Results, pp6-9
Descriptive data	14	(a) Give characteristics of study participants (*e.g.*, demographic, clinical, social) and information on exposures and potential confounders<br>(b) Indicate the number of participants with missing data for each variable of interest<br>(c) *Cohort study* - summarise follow-up time (*e.g.*, average and total amount)	Results, pp6-9		
Outcome data	15	*Cohort study* - Report numbers of outcome events or summary measures over time<br>*Case-control study* - Report numbers in each exposure	Results, pp6-9		

For peer review only

		category, or summary measures of exposure<br>*Cross-sectional study* - Report numbers of outcome events or summary measures			
Main results	16	(a) Give unadjusted estimates and, if applicable, confounder-adjusted estimates and their precision (e.g., 95% confidence interval). Make clear which confounders were adjusted for and why they were included<br>(b) Report category boundaries when continuous variables were categorized<br>(c) If relevant, consider translating estimates of relative risk into absolute risk for a meaningful time period	Results, pp6-9		
Other analyses	17	Report other analyses done—e.g., analyses of subgroups and interactions, and sensitivity analyses	Results, pp6-9		
**Discussion**					
Key results	18	Summarise key results with reference to study objectives	Discussion, pp10-11		
Limitations	19	Discuss limitations of the study, taking into account sources of potential bias or imprecision. Discuss both direction and magnitude of any potential bias	Discussion, pp10-11	RECORD 19.1: Discuss the implications of using data that were not created or collected to answer the specific research question(s). Include discussion of misclassification bias, unmeasured confounding, missing data, and changing eligibility over time, as they pertain to the study being reported.	Discussion, pp10-11
Interpretation	20	Give a cautious overall interpretation of results considering objectives,	Discussion, pp10-11		

For peer review only

		limitations, multiplicity of analyses, results from similar studies, and other relevant evidence			
Generalisability	21	Discuss the generalisability (external validity) of the study results	Discussion, pp10-11		
**Other Information**					
Funding	22	Give the source of funding and the role of the funders for the present study and, if applicable, for the original study on which the present article is based	No study-specific funding		
Accessibility of protocol, raw data, and programming code		..	Methods, pp4-5 and appendix 1	RECORD 22.1: Authors should provide information on how to access any supplemental information such as the study protocol, raw data, or programming code.	Corresponding author details

*Reference: Benchimol EI, Smeeth L, Guttmann A, Harron K, Moher D, Petersen I, Sørensen HT, von Elm E, Langan SM, the RECORD Working Committee. The REporting of studies Conducted using Observational Routinely-collected health Data (RECORD) Statement. *PLoS Medicine* 2015; in press.

*Checklist is protected under Creative Commons Attribution (CC BY) license.

For peer review only

BMJ Open

Numbers and types of neurological emergencies in England and the influence of socio-economic deprivation: a retrospective analysis of Hospital Episode Statistics data

Journal:	*BMJ Open*
Manuscript ID	bmjopen-2022-061843.R1
Article Type:	Original research
Date Submitted by the Author:	22-Jul-2022
Complete List of Authors:	Jackson, Michael; United Kingdom Department of Health and Social Care, Office for Health Improvement and Disparities (OHID)<br>Szczepaniak, Marta; United Kingdom Department of Health and Social Care, Office for Health Improvement and Disparities (OHID)<br>Wall, Jasmine; Lancaster University; Lancashire Teaching Hospitals NHS Foundation Trust<br>Maskery, Mark; Lancaster University; Lancashire Teaching Hospitals NHS Foundation Trust<br>Mummery, Catherine; University College London Hospitals NHS Foundation Trust<br>Morrish, Paul; Great Western Hospital Foundation NHS Trust<br>Williams, Adrian; University Hospitals Birmingham NHS Foundation Trust; NHS England<br>Knight, Joanne; Lancaster University<br>Emsley, Hedley; Lancaster University; Lancashire Teaching Hospitals NHS Foundation Trust
**Primary Subject Heading**:	Neurology
Secondary Subject Heading:	Epidemiology
Keywords:	Epilepsy < NEUROLOGY, Neurology < INTERNAL MEDICINE, Public health < INFECTIOUS DISEASES

SCHOLARONE™
Manuscripts

I, the Submitting Author has the right to grant and does grant on behalf of all authors of the Work (as defined in the below author licence), an exclusive licence and/or a non-exclusive licence for contributions from authors who are: i) UK Crown employees; ii) where BMJ has agreed a CC-BY licence shall apply, and/or iii) in accordance with the terms applicable for US Federal Government officers or employees acting as part of their official duties; on a worldwide, perpetual, irrevocable, royalty-free basis to BMJ Publishing Group Ltd ("BMJ") its licensees and where the relevant Journal is co-owned by BMJ to the co-owners of the Journal, to publish the Work in this journal and any other BMJ products and to exploit all rights, as set out in our licence.

The Submitting Author accepts and understands that any supply made under these terms is made by BMJ to the Submitting Author unless you are acting as an employee on behalf of your employer or a postgraduate student of an affiliated institution which is paying any applicable article publishing charge ("APC") for Open Access articles. Where the Submitting Author wishes to make the Work available on an Open Access basis (and intends to pay the relevant APC), the terms of reuse of such Open Access shall be governed by a Creative Commons licence – details of these licences and which Creative Commons licence will apply to this Work are set out in our licence referred to above.

Other than as permitted in any relevant BMJ Author's Self Archiving Policies, I confirm this Work has not been accepted for publication elsewhere, is not being considered for publication elsewhere and does not duplicate material already published. I confirm all authors consent to publication of this Work and authorise the granting of this licence.

For peer review only

Numbers and types of neurological emergencies in England and the influence of socio-economic deprivation: a retrospective analysis of Hospital Episode Statistics data

A retrospective empirical research study

Jackson M[1], Szczepaniak M[1], Wall J[2,3], Maskery M[2,3], Mummery C[4], Morrish P[5], Williams A[6,7], Knight J[1,2], Emsley HCA[1,2,3]

[1]Neurology & Dementia Intelligence team part of Public Health Analysis Group, Office for Health Improvement and Disparities (OHID), Department of Health and Social Care, UK (Formerly Public Health England); [2]Lancaster Medical School, Lancaster University, Lancaster, UK; [3]Lancashire Teaching Hospitals NHS Foundation Trust, Preston, UK; [4]Dementia Research Centre, National Hospital for Neurology and Neurosurgery, University College London Hospitals NHS Foundation Trust, London, UK; [5]Greater Western Hospitals NHS Foundation Trust, Swindon, UK; [6]University Hospitals Birmingham NHS Foundation Trust, Birmingham, UK; [7]Neuroscience Clinical Reference Group, NHS England, London, UK

Author email addresses

Michael Jackson	michael.jackson@dhsc.gov.uk
Marta Szczepaniak	marta.szczepaniak@dhsc.gov.uk
Jasmine Wall	jasmine.wall@lthtr.nhs.uk
Mark Maskery	mark.maskery@lthtr.nhs.uk
Cath Mummery	cath.mummery@nhs.net
Paul Morrish	p.morrish@nhs.net
Adrian Williams	adrian.williams@uhb.nhs.uk
Jo Knight	jo.knight@lancaster.ac.uk
Hedley Emsley*	hedley.emsley@lancaster.ac.uk

*corresponding author address. Department of Neurology, Royal Preston Hospital, Lancashire Teaching Hospitals NHS Foundation Trust, Sharoe Green Lane, Preston, PR2 9HT

For peer review only

Abstract

Objectives In this first large-scale analysis of neurological emergency admissions in England, we determine the number and types of emergency admissions with neurological emergency diagnostic codes, how many are under the care of a neurologist or neurosurgeon, and how such admissions vary by levels of deprivation

Design Retrospective empirical research employing a derived list of neurological emergency diagnostic codes

Setting This study used the Hospital Episode Statistics (HES) dataset for the financial year 2019/2020 based on 17 million in-year inpatient admissions in England including 6.5 million (100%) emergency admissions with any diagnosis codes

Results There were 1.4 million (21.2%) emergency inpatient admissions with a mention of any neurological code, approx. 248,455 (3.8%) with mention of a specific neurological emergency code from the derived list, and 72,485 (1.1%) included such a code as the primary reason for admission. The highest number of in-year admissions for adults was for epilepsy (145,995), with epilepsy as the primary diagnostic code in 15,945 (10.9%). Acute nerve root/spinal cord syndrome (41,215), head injury (29,235) and subarachnoid haemorrhage (18,505) accounted for the next three highest number of admissions. 3,230 (1.4%) in-year emergency hospital admissions with mention of a neurological emergency code were under the care of a neurologist or neurosurgeon, with only 1,315 (0.9%) admissions with mention of an epilepsy code under a neurologist. There was significant variation for epilepsy and functional neurological disorders (FNDs) in particular by Index of Multiple Deprivation decile. The association between deprivation and epilepsy and FND was significant with p-values of 2.5e-6, 1.5e-8 respectively.

Conclusions This study has identified important findings in relation to the burden of neurological emergency admissions but further work is needed, with greater clinical engagement in diagnostic coding, to better understand the implications for workforce and changes to service delivery needing to be implemented.

For peer review only

Strengths and limitations

- First analysis of its type arising from an academic partnership with the Office for Health Improvement and Disparities, utilising Hospital Episode Statistics data
- Large-scale analysis based on 6.5 million emergency admissions increases robustness of findings
- Pragmatic approach to creation of list defining neurological emergencies
- Retrospective nature of study limits interpretation of some findings such as the nature of suspected cauda equina presentations
- Inclusion of emergency admissions with mention of neurological condition rather than solely neurological emergencies *per se* on account of coding accuracy concerns

For peer review only

Introduction

Medical emergency admissions continue to rise across the UK. They are more than twice as likely among the most deprived population decile by comparison with mean deprivation, reinforcing the point that challenges are social as well as medical[1,2]. Many emergency admissions to hospital are considered to be avoidable and patients often stay in hospital longer than is necessary[3]. Emergency admissions are also disruptive of elective care delivery, prolonging waiting times. Hospital Episodes Statistics (HES) data for 2016/17 suggest that approximately 18% of medical emergency admissions included a mention of neurological conditions including stroke and dementia, totalling 1.09 million, a rise of 21% over the previous 5 years[4]. Excess mortality has also been reported among patients with epilepsy[5]. At a time when costs are rising and resources are increasingly under pressure, it is all the more important to have a better understanding of neurological emergency admissions and underlying factors such as social deprivation.

There has been little by way of systematic evaluation of data pertaining to neurological emergency admissions. Previous work has used a much broader definition, counting neurological conditions present in patients admitted as an emergency, including stroke and dementia, rather than neurological emergencies *per se*. The present work has by necessity involved the creation of a list defining neurological emergencies. Much remains unknown about neurological emergency admissions, including their total number, the distribution of neurological conditions, and the effect of socio-economic deprivation. There have been some studies investigating the influence of comorbidities and race in the United States [6,7]. This study represents the first large scale analysis of neurological emergency admissions in England, and the first OHID-academic partnership in neurology. For the first time, the data provide some insight into the number and types of emergency admissions with neurological emergency codes, the number of such admissions under the care of a neurologist, and how such admissions vary on the basis of factors such as levels of deprivation.

For peer review only

Methods

Defining neurological emergencies

For the purposes of this work it was necessary to create a list of conditions typically regarded as neurological emergencies, and to map these to the relevant ICD-10 codes. An initial set of neurological conditions was proposed by AW as the basis for the study, comprising causes of acute paralysis such as Guillain-Barré syndrome, myasthenic crisis, encephalitis, spinal cord compression and status epilepticus. The ICD-10 codes most closely matching these 'index' entities were compiled into a list. Other ICD-10 codes in close hierarchical proximity to the 'index' ICD-10 codes were added from a previously published Public Health England (PHE) neurology code collection [8], which comprised 581 4-digit ICD-10 codes for 19 condition groups. The 'long list' of candidate neurological emergency codes, consisting of 136 ICD-10 codes, was reviewed iteratively by the project team and clinicians working alongside, and refined into a short list of 96 4-digit ICD-10 codes (appendix 1) for use in the work reported here, subsequently referred to as the 'derived list' [of neurological emergency codes]. The selection process used clinical judgement to decide which conditions or groups of conditions to include or exclude. Of particular note, stroke, despite being a classical neurological emergency, was excluded from this exercise given that stroke is already the subject of the existing national audit system (SSNAP). Codes for conditions presenting with secondary headache have been included; codes for primary headache disorders have not been included in the present work. Also of note was the decision to include functional neurological disorders (FNDs) on the basis that we believed these to contribute significantly to the total number of emergency hospital admissions for neurological conditions, as well as the need for patients with FND to be referred to specialists with expertise in neurological diagnosis [9]. FNDs is a term given to neurological features that are not due to any physical neurological disorder. We have defined relevant terminology in Box 1.

Defining study group

The hospital activity included in this study were admissions to hospital occurring during the financial year 2019/2020 in England – described as an in-year admission, where the admission was classed as an emergency (non-elective) admission to hospital and where the selected neurological emergency codes are in any diagnosis position, on any consultant episode during the hospital stay.

Data source, data extraction and statistics

The data source used in this study is the Admitted Patient Care version of the Hospital Episode Statistics dataset for the financial year 2019/2020. Data extraction was undertaken by the OHID Neurology Dementia Intelligence team using Structured Query Language (SQL) with further data manipulation, including descriptive statistics, using Microsoft Excel. Counts and percentages were extracted and/or calculated for various categories, confidence intervals were estimated and data was visualised in a range of chart types. Other than for the data in table 1 and the associated summary text, the paediatric population was excluded from further analyses. Chi squared tests were used to determine if the distribution of admission by primary code versus secondary was independent of diagnosis and to assess the independence of diagnosis with involvement of a neurologist at admission, later in the spell or never. A Cramérs V was used to assess independence of admission with indices of deprivation; this was stratified by a range of diagnosis type. For the first two analyses a code assigned to admission is the unit of analysis, for the third analysis the unit of analysis is admission of an individual.

For peer review only

Patient and public involvement

This was a retrospective empirical research study based on a secondary analysis of routinely collected anonymised clinical data. Patient and public involvement was not applicable.

Box 1. Relevant terminology

People admitted to hospital will always have a responsible medical officer (consultant) assigned for the time under that consultant, or episode (**consultant episode**).

The consultant episode at the start of the hospital stay is the **admission episode**; the episode at the end of the hospital spell is called the **discharge episode**.

Hospital admissions are the start of **hospital spells**, which comprise one or more consultant episodes.

ICD-10 – International Statistical Classification of Diseases and Related Health Problems (ICD) (10th edition) codes, or **hospital diagnosis codes**. ICD-10 is a legally mandated health data standard.

Conditions present during the consultant episode can be described using up to 20 corresponding ICD-10 codes . Hospital stays with multiple consultant episodes can have very differing collections of ICD-10 codes recorded on each episode.

The ICD-10 code that is recorded in the first position on a consultant episode is known as the **primary diagnosis**. Subsequent codes on the same episodes are considered **secondary diagnoses** and may represent conditions of lesser importance for this particular consultant episode or other long-term conditions/co-morbidities of the patient.

Hospital episode statistics (HES) are financial year based. Stays in hospital can cross the financial year timelines, therefore the number of admissions and discharges in any one financial year may not be equal. **In-year hospital admissions** relates to the count of all hospital stays that start in the financial year. **In-year hospital discharges** relate to the count of all hospital stays that end during the financial year. **HES ID** is a pseudonymised unique identifier for each individual patient, which can be used to group hospital episodes and spells by individual

Results

Emergency inpatient admissions with any neurological codes or neurological emergency codes

Table 1 shows totals for inpatient activity for all ages by admission type for 2019/2020. Of a total of over 17 million inpatient admissions, 6.5 million were emergency admissions. Among these, 1.4 million (21%) were emergency inpatient admissions with a mention of any neurological code (including dementia and stroke). Emergency inpatient admissions with a specific neurological emergency code from the derived list (appendix 1) represented 3.8% of all emergency inpatient admissions, and such a code was the primary reason for admission in 1.1% of all emergency inpatient admissions. Among all emergency adult in-year admissions by spell with specific neurological diagnosis codes from the derived list 132,485 (54.4%) comprised a single episode.

The number of unique adult patients (using HESID as a proxy) with in-year admissions by spell with specific neurological diagnosis codes from the derived list was 167,735. 75.6% (126,800) of these individuals have only one admission.

Table 1: Inpatient hospital activity by admission type; all ages; England; 2019/20

	Counts	Percentage of all emergency inpatient admissions
All inpatient admissions: in-year admissions by spell with any diagnosis codes	17,183,220	NA
Emergency inpatient admissions: in-year admissions by spell with any diagnosis codes	6,506,970	100%
Emergency inpatient admissions with a mention of any neurological code (including dementia and stroke): in-year admissions by spell	1,380,975	21.2%
Emergency inpatient admissions with neurological emergency codes: in-year admissions by spell with specific neurological diagnosis codes	248,455	3.8%
Emergency inpatient admissions with neurological emergency code as primary reason for admission: in-year admissions by spell with specific neurological diagnosis codes	72,485	1.1%

Source: OHID Neurology Dementia Intelligence using Hospital Episode Statistics Admited Patient Care, NHS Digital

For peer review only

Neurological conditions

Table 2 shows adult emergency inpatient hospital admissions with a mention of neurological emergency codes. Among a total of 237,755 such admissions, 65,060 (27.4%) had a neurological emergency code as a primary diagnostic code on the starting episode. Similar percentages were noted for acute paralysis (24.5%), functional disorders (29.8%), acute nerve root/cord syndrome (30.7%) and encephalitis (34.2%).

However we see a strikingly different pattern for epilepsy, subarachnoid haemorrhage (SAH) and head injury (HI). The highest number of in-year admissions was for epilepsy (145,995), yet the percentage of those with epilepsy as the primary diagnostic code was only 10.9%. By comparison, the primary diagnostic codes for head injury appeared in 73.1% of 29,235 admissions for this condition, and similarly for subarachnoid haemorrhage the primary diagnostic code appeared in 57.9% of 18,505 admissions. The p-value of the association of diagnosis with primary code is $<10^{-6}$.

Table 2: Emergency inpatient hospital admissions with a mention of neurological emergency codes; adults aged 18 years and over; England; 2019/20

	In-year admissions	In-year admissions with primary diagnosis on starting episode	Primary diagnosis as % of in-year admissions
neurological emergencies	237,755	65,060	27.4%
emergency acute nerve root/cord syndrome	41,215	12,655	30.7%
emergency acute paralysis	2,600	635	24.5%
emergency encephalitis	5,240	1,790	34.2%
emergency epilepsy	145,995	15,945	10.9%
emergency functional disorders	6,545	1,950	29.8%
emergency head injuries	29,235	21,365	73.1%
emergency subarachnoid haemorrhage	18,505	10,720	57.9%

Source: OHID Neurology Dementia Intelligence using Hospital Episode Statistics Admited Patient Care, NHS Digital

For peer review only

Proportion of admissions under a neurologist

Figure 1 shows the percentage of in-year adult emergency hospital admissions with mention of a neurological emergency code under the care of a consultant in neurology or neurosurgery. Overall, among 237,755 such admissions, 3,230 (1.4%) were under the care of a consultant neurologist and 10,760 (4.5%) were under the care of a consultant neurosurgeon, with the majority of the admissions under a neurosurgeon having SAH or HI diagnostic codes (see supplementary table 1). Only 1,315 (0.9%) of admissions with mention of an epilepsy diagnostic code were under the care of a consultant neurologist. The p-value of the association of diagnosis with care under a neurologist either at admission/later in the spell or not at all is $<10^{-6}$.

Influence of socio-economic deprivation

Figure 2 shows a proxy for the number of people admitted (total, functional, epilepsy, SAH) by deciles of deprivation using the Indices of Multiple Deprivation [10] (see also supplementary table 2). This shows that the number of people admitted varies significantly by social deprivation. This pattern is seen for the total admissions with mention of neurological emergency codes, but is more apparent for epilepsy and is most striking for functional neurological disorders, where the greatest number were in the most deprived and the fewest in the least deprived (p-values 1.6e-4, 2.5e-6, 1.5e-8 respectively). By comparison, admissions with mention of SAH did not exhibit much variation by social deprivation (p-value=0.01).

For peer review only

Discussion

In this first large-scale analysis of 1.4 million emergency inpatient admissions in England for 2019/2020 with a mention of any neurological code, there were 248,455 with mention of a specific neurological emergency code from the derived list. The highest number was for epilepsy (145,995) with the next three most frequent causes being acute nerve root/spinal cord syndrome, head injury and subarachnoid haemorrhage. 1.4% of admissions with mention of a neurological emergency code were under a neurologist or neurosurgeon, with only 0.9% of those with mention of an epilepsy code under a neurologist. The greatest number of admissions were in the most deprived, particularly for epilepsy and functional neurological disorders. Being able to determine the proportion of emergency admissions that constitute neurological emergency presentations is clearly crucial for service and clinical pathway design and workforce planning.

It was necessary to formulate a list of neurological emergencies and associated list of ICD-10 codes as a prelude to the analyses in this study. We took a pragmatic approach based on current UK practice, taking into account factors such as existing well defined pathways of care (eg acute stroke) or the absence of well defined pathways (eg FND). Although perhaps not classically considered neurological emergencies, FND presentations are increasingly common. This is with respect to their impact both the individual and upon healthcare resources, resources which are often used inappropriately and not in alignment with the needs of such patients.

We found that among adult emergency admissions with mention of a neurological emergency code, this was the primary diagnostic code in 27.4% overall. However, despite epilepsy accounting for the greatest number of admissions in our dataset, epilepsy appeared as the primary diagnostic code in only 10.9% by comparison with 73.1% for head injury 57.9% for SAH. It is important to note that epilepsy codes have been included among the neurological emergency codes but may reflect epilepsy as a comorbidity and not the reason for emergency admission, hence the comparatively low proportion of patients with epilepsy codes as the primary diagnostic code. A proportion of patients assigned epilepsy codes may have had dissociative seizures (DS). Admissions with mention of epilepsy codes merit further analysis in future work, particularly given recent data on epilepsy related mortality [5]. The current analysis cannot explain the differences between conditions in the proportion of relevant primary diagnostic codes being assigned. However, it is conceivable that the high proportion of head injury admissions with head injury as the primary diagnostic code, and similarly for SAH, may reflect the readily apparent the relative ease and pace of establishing the primary reason for admission.

We found that among emergency admissions with mention of a neurological emergency code, 1.4% were under the care of a consultant neurologist and 4.5% were under the care of a consultant neurosurgeon; only 0.9% with mention of an epilepsy diagnostic code were under the care of a consultant neurologist. Many admissions with single episodes not under neurology or neurosurgery also suggests many patients may not receive input from these disciplines despite having neurological codes assigned. Although the percentage of admissions under a consultant neurologist may appear disproportionately low given the greater number of neurologists by comparison with neurosurgeons, this may partly reflect the different balance of commitments between outpatient and inpatient pathways between neurology and neurosurgery. These findings are in keeping with previous data suggesting that a low proportion of epilepsy emergency admissions are under a neurologist. Further work, such as the prospective capture of neurological emergency codes with greater clinical engagement to improve their accuracy, is needed, but our findings are in general agreement with the recognised urgent need to address workforce capacity to support neurological emergency admissions.

For peer review only

The present study highlights additional concerns, particularly the apparent relationship between admissions for FND and socioeconomic deprivation. We know that in many areas in England there is a significant gap in terms of provision of appropriate services for patients with FND [11], very likely to increase the likelihood of re-presentation as an emergency. A better understanding of emergency admissions for FND is needed to determine how clinical pathways can be improved for such patients. The retrospective nature of the present study limits interpretation of findings such as acute nerve root/spinal cord syndrome being arguably more frequent than expected; one possibility is a rise in ‘scan-negative’ cauda equina syndrome, itself another potential FND[9].

An important limitation of the methodology employed by this study is the inclusion of emergency admissions which mention neurological conditions, ie admissions which include codes deemed, as part of this study, relevant for neurological emergencies. These codes could appear at any position in the patient’s diagnostic coding, and during any episode(s) comprising the given spell; implicit in this approach is the possibility that the neurological condition was not the reason for the emergency admission. This, of course, is different to the inclusion of neurological emergencies *per se*, where the primary cause of the emergency admission was neurological. Previous work has raised concern about the accuracy of current data collection methods, including the erroneous assignment of neurological codes [12].

Conclusion We have for the first time quantified the number of neurological emergency admissions across England, the distribution of conditions, the proportion under the care of a neurologist or neurosurgeon and evidence of variation by social deprivation. Further work, including improved clinical engagement with coding to ensure data accuracy, is required to understand the drivers of these findings. Further analyses are also needed to investigate issues such as the effect of hospital setting (eg district general hospital vs neurosciences centre), the point in the spell at which the neurological diagnosis is made and the influence of neurologist involvement. These activities will inform clinical pathway change across primary and secondary care to reduce avoidable admissions and improve care.

For peer review only

Contributorship statement

MJ, MS, JK and HE conceived and designed the work and jointly undertook the analysis and interpretation of the data. MJ, JK and HE jointly drafted the manuscript and revised it critically for important intellectual content. AW contributed to the conception of the work. JW, MM, CM, PM and AW contributed to the development of the derived list of neurological emergency diagnostic codes. All authors approved the final version.

The corresponding author attests that all listed authors meet authorship criteria and that no others meeting the criteria have been omitted.

Competing interests

The authors have no relevant competing interests to declare.

Funding

This study received no specific funding.

Data availability statement

Information relating to access to HES data used for this study can be found at https://digital.nhs.uk/data-and-information/data-tools-and-services/data-services/hospital-episode-statistics/users-uses-and-access-to-hospital-episode-statistics.

Ethics statement

Ethics Committee approval was not required as the research presented in this manuscript comprises a secondary analysis of routinely collected anonymised clinical data.

For peer review only

References

1. McCormick B, Hill P-S, Poteliakhoff E. 2012. Are hospital services used differently in deprived areas? Evidence to identify commissioning challenges. http://chseo.org.uk/downloads/wp2-hospitalservices-deprivedareas.pdf (accessed December 2021)
2. Purdy S. 2010. Avoiding hospital admissions. London: The King's Fund. https://www.kingsfund.org.uk/sites/default/files/Avoiding-Hospital-Admissions-Sarah-Purdy-December2010.pdf (accessed January 2022)
3. National Audit Office. 2013. Emergency admissions to hospital: managing the demand. https://www.nao.org.uk/report/emergency-admissions-hospitals-managing-demand/ (accessed December 2021)
4. The Neurological Alliance. 2019. Neuro Numbers 2019. https://www.neural.org.uk/assets/pdfs/neuro-numbers-2019.pdf (accessed December 2021)
5. Public Health England. 2018. Deaths associated with neurological conditions data briefing. https://www.gov.uk/government/publications/deaths-associated-with-neurological-conditions (accessed January 2022)
6. Sajatovic M, Welter E, Tatsuoka C, Perzynski AT, Einstadter D. Electronic medical record analysis of emergency room visits and hospitalizations in individuals with epilepsy and mental illness comorbidity. Epilepsy Behav. 2015 Sep;50:55-60. doi: 10.1016/j.yebeh.2015.05.016. Epub 2015 Jun 25. PMID: 26119621; PMCID: PMC4793963.
7. Fantaneanu TA, Hurwitz S, van Meurs K, Llewellyn N, O'Laughlin KN, Dworetzky BA. Racial differences in Emergency Department visits for seizures. Seizure. 2016 Aug;40:52-6. doi: 10.1016/j.seizure.2016.06.007. Epub 2016 Jun 16. PMID: 27344498.
8. Public Health England. 2021. Neurology and Dementia Intelligence: ICD10 code collections. https://fingertips.phe.org.uk/profile-group/mental-health/supporting-information/ndi-code-collections (accessed December 2021)
9. Bennett K, Diamond C, Hoeritzauer I, Gardiner P, McWhirter L, Carson A, Stone J. A practical review of functional neurological disorder (FND) for the general physician. Clin Med (Lond). 2021 Jan;21(1):28-36. doi: 10.7861/clinmed.2020-0987. PMID: 33479065; PMCID: PMC7850207.
10. Ministry of Housing, Communities & Local Government. 2019. English indices of deprivation 2019. https://www.gov.uk/government/statistics/english-indices-of-deprivation-2019 (accessed December 2021)
11. O'Keeffe S, Chowdhury I, Sinanaj A, Ewang I, Blain C, Teodoro T, Edwards M, Yogarajah M. A Service Evaluation of the Experiences of Patients With Functional Neurological Disorders Within the NHS. Front Neurol. 2021 May 31;12:656466. doi: 10.3389/fneur.2021.656466. PMID: 34135848; PMCID: PMC8200476.
12. Kanagaratnam M, Boodhoo A, MacDonald BK, Nitkunan A. Prevalence of acute neurology: a 2-week snapshot in a district general hospital. Clin Med (Lond). 2020;20(2):169-173. doi:10.7861/clinmed.2019-0377

Figure Legends

Figure 1. Percentage of in-year emergency hospital admissions with mention of neurological emergency codes admitted under the care of a consultant neurologist or neurosurgeon; adults aged 18 years and over; England; 2019/20; source: OHID Neurology Dementia Intelligence using Hospital Episode Statistics Admitted Patient Care, NHS Digital.

[see separate figure 1 file]

Figure 2. Proportion of unique HESIDs (proxy for individuals) with an in-year neurological emergency hospital admission, by decile of deprivation and condition group; adults aged 18 years and over; Indices of Multiple Deprivation 2019; England; 2019/20; source: OHID Neurology Dementia Intelligence using Hospital Episode Statistics Admitted Patient Care, NHS Digital.

[see separate figure 2 file]

For peer review only

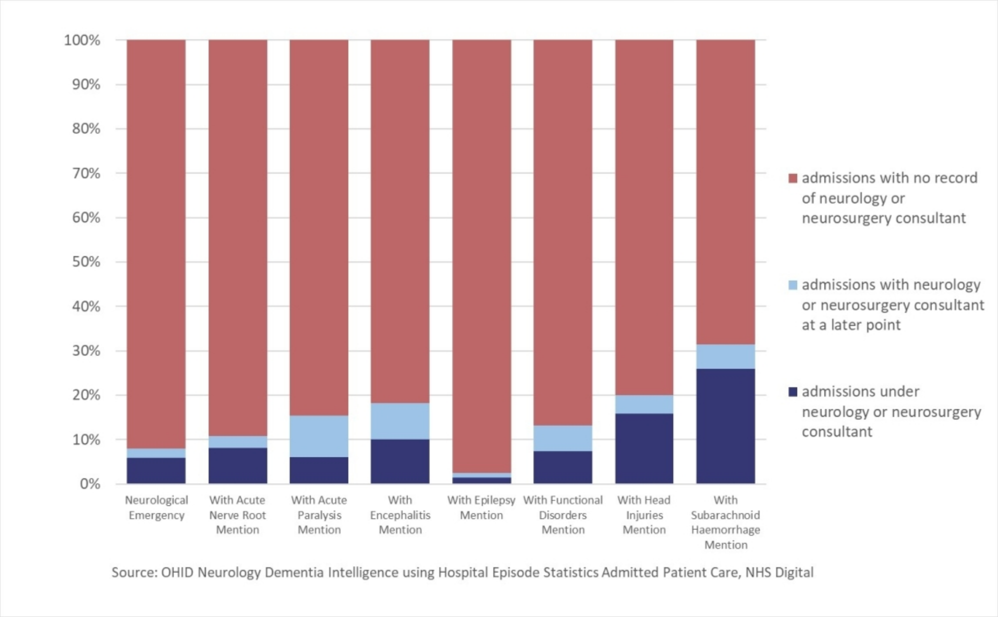

Figure 1. Percentage of in-year emergency hospital admissions with mention of neurological emergency codes admitted under the care of a consultant neurologist or neurosurgeon; adults aged 18 years and over; England; 2019/20; source: OHID Neurology Dementia Intelligence using Hospital Episode Statistics Admitted Patient Care, NHS Digital.

256x159mm (150 x 150 DPI)

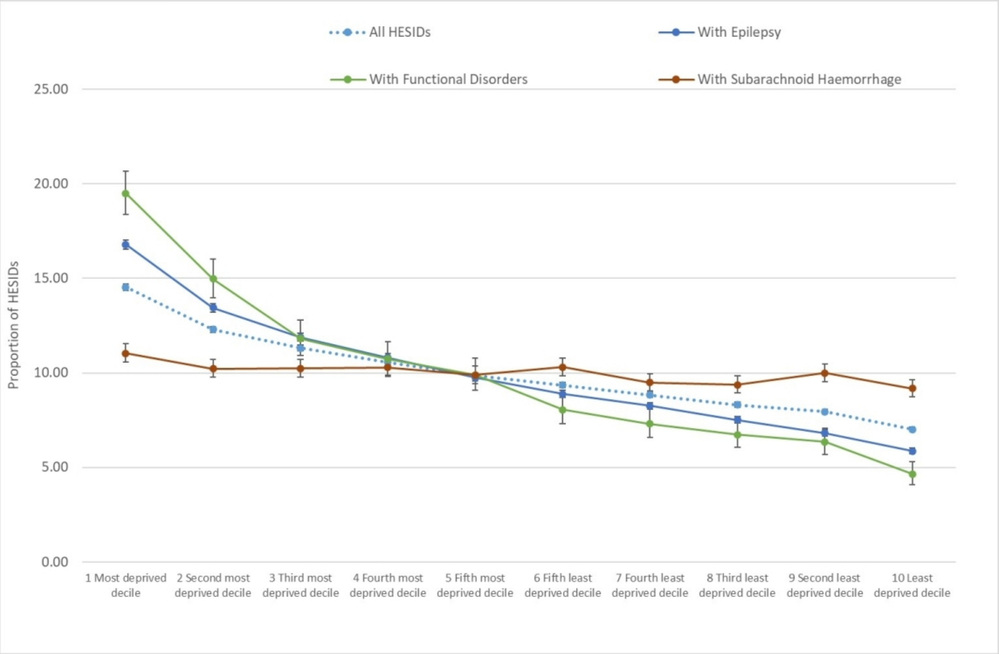

Figure 2. Proportion of unique HESIDs (proxy for individuals) with an in-year neurological emergency hospital admission, by decile of deprivation and condition group; adults aged 18 years and over; Indices of Multiple Deprivation 2019; England; 2019/20; source: OHID Neurology Dementia Intelligence using Hospital Episode Statistics Admitted Patient Care, NHS Digital.

258x168mm (150 x 150 DPI)

Supplementary Table 1: Percentage of in-year emergency hospital admissions with mention of neurological emergency codes admitted under the care of a consultant neurologist or neurosurgeon; adults aged 18 years and over; England; 2019/20

	Neurological Emergency	With Acute Nerve Root Mention	With Acute Paralysis Mention	With Encephalitis Mention	With Epilepsy Mention	With Functional Disorders Mention	With Head Injuries Mention	With Subarachnoid Haemorrhage Mention
admissions under neurology consultant	3,230	545	145	270	1,315	445	305	440
%	*1.36%*	*1.32%*	*5.58%*	*5.19%*	*0.90%*	*6.80%*	*1.04%*	*2.38%*
admissions under neurosurgery consultant	10,760	2,905	10	255	810	40	4,360	4,375
%	*4.52%*	*7.05%*	*0.42%*	*4.89%*	*0.56%*	*0.63%*	*14.91%*	*23.63%*
admissions under neurology or neurosurgery consultant	13,860	3,355	155	525	2,120	485	4,655	4,800
%	*5.83%*	*8.15%*	*6.00%*	*10.06%*	*1.45%*	*7.40%*	*15.92%*	*25.95%*
admissions with neurology consultant at a later point	3,150	435	250	410	1,355	370	390	320

For peer review only

%	*1.32%*	*1.06%*	*9.58%*	*7.81%*	*0.93%*	*5.67%*	*1.33%*	*1.72%*
admissions with neurosurgery consultant at a later point	2,265	795	*	55	165	10	945	835
%	*0.95%*	*1.93%*	*0.00%*	*1.07%*	*0.11%*	*0.17%*	*3.23%*	*4.52%*
admissions with neurology or neurosurgery consultant at a later point	5,000	1,110	245	430	1,465	375	1,200	1,010
%	*2.10%*	*2.70%*	*9.35%*	*8.21%*	*1.00%*	*5.72%*	*4.11%*	*5.49%*
admissions with no record of neurology or neurosurgery consultant	218,890	36,745	2,200	4,280	142,410	5,690	23,380	12,690
%	*92.07%*	*89.15%*	*84.64%*	*81.73%*	*97.54%*	*86.89%*	*79.97%*	*68.56%*
**in year admissions**	**237,755**	**41,215**	**2,600**	**5,240**	**145,995**	**6,545**	**29,235**	**18,505**
Source: OHID Neurology Dementia Intelligence using Hospital Episode Statistics Admited Patient Care, NHS Digital								

For peer review only

Supplementary Table 2

Deciles of Deprivation England		All HESIDs	With Epilepsy	With Functional Disorders	With Subarachnoid Haemorrhage
**1 Most deprived**	Count	23,600	14,970	910	1,770
	Percentage	14.5%	16.8%	19.5%	11.0%
**2**	Count	19,965	11,990	700	1,640
	Percentage	12.3%	13.4%	15.0%	10.2%
**3**	Count	18,365	10,595	555	1,640
	Percentage	11.3%	11.9%	11.8%	10.2%
**4**	Count	17,175	9,640	500	1,650
	Percentage	10.6%	10.8%	10.7%	10.3%
**5**	Count	16,005	8,700	465	1,590
	Percentage	9.9%	9.8%	9.9%	9.9%
**6**	Count	15,190	7,920	375	1,655
	Percentage	9.4%	8.9%	8.1%	10.3%
**7**	Count	14,340	7,365	340	1,525
	Percentage	8.8%	8.3%	7.3%	9.5%
**8**	Count	13,510	6,700	315	1,505
	Percentage	8.3%	7.5%	6.7%	9.4%
**9**	Count	12,920	6,080	300	1,605
	Percentage	8.0%	6.8%	6.3%	10.0%
**10 Least deprived**	Count	11,375	5,225	220	1,470
	Percentage	7.0%	5.9%	4.7%	9.2%

Source: OHID Neurology Dementia Intelligence using Hospital Episode Statistics Admitted Patient Care, NHS Digital

For peer review only

Appendix 1

Neurological Emergency ICD10 codes – 96 codes (short list)

NeuroEmergencyConditionGroup	NeuroEmergencyICD10CodeHES	ICD10_Level3Header
Acute paralysis	A521	Symptomatic neurosyphilis
Encephalitis	A830	Japanese encephalitis
Encephalitis	A831	Western equine encephalitis
Encephalitis	A832	Eastern equine encephalitis
Encephalitis	A833	St Louis encephalitis
Encephalitis	A834	Australian encephalitis
Encephalitis	A835	California encephalitis
Encephalitis	A836	Rocio virus disease
Encephalitis	A838	Other mosquito-borne viral encephalitis
Encephalitis	A839	Mosquito-borne viral encephalitis
Encephalitis	A840	Far Eastern tick-borne encephalitis [Russian spring-summer encephalitis]
Encephalitis	A841	Central European tick-borne encephalitis
Encephalitis	A848	Other tick-borne viral encephalitis
Encephalitis	A849	Tick-borne viral encephalitis
Encephalitis	A850	Enteroviral encephalitis
Encephalitis	A851	Adenoviral encephalitis
Encephalitis	A852	Arthropod-borne viral encephalitis, unspecified
Encephalitis	A858	Other specified viral encephalitis

Encephalitis	A86X	Unspecified viral encephalitis
Functional Disorders	F440	Dissociative amnesia
Functional Disorders	F441	Dissociative fugue
Functional Disorders	F442	Dissociative stupor
Functional Disorders	F443	Trance and possession disorders
Functional Disorders	F444	Dissociative motor disorders
Functional Disorders	F445	Dissociative convulsions
Functional Disorders	F446	Dissociative anaesthesia and sensory loss
Functional Disorders	F447	Mixed dissociative conversion disorders
Functional Disorders	F448	Other dissociative conversion disorders
Functional Disorders	F449	Dissociative conversion disorder unspecified
Encephalitis	G040	Acute disseminated encephalitis
Encephalitis	G041	Tropical spastic paraplegia
Encephalitis	G042	Bacterial meningoencephalitis and meningomyelitis NEC
Encephalitis	G048	Other encephalitis myelitis and encephalomyelitis
Encephalitis	G049	Encephalitis myelitis and encephalomyelitis unspecified
Encephalitis	G050	Encephalitis myelitis and encephalomyelitis in bacterial diseases classified elsewhere
Encephalitis	G051	Encephalitis myelitis and encephalomyelitis in viral diseases classified elsewhere
Encephalitis	G052	Encephalitis myelitis and encephalomyelitis in other infectious and parasitic diseases classified elsewhere
Encephalitis	G058	Encephalitis myelitis and encephalomyelitis in other diseases classified elsewhere
Acute nerve root/cord syndrome	G114	Hereditary spastic paraplegia
Epilepsy	G409	Epilepsy unspecified
Epilepsy	G410	Grand mal status epilepticus
Epilepsy	G411	Petit mal status epilepticus

For peer review only

Epilepsy	G412	Complex partial status epilepticus
Epilepsy	G418	Other status epilepticus
Epilepsy	G419	Status epilepticus unspecified
Acute nerve root/cord syndrome	G549	Nerve root and plexus disorder
Acute nerve root/cord syndrome	G551	Nerve root and plexus compressions in intervertebral disc disorders
Acute paralysis	G610	Guillain-Barre syndrome
Acute nerve root/cord syndrome	G820	Flaccid paraplegia
Acute nerve root/cord syndrome	G821	Spastic paraplegia
Acute nerve root/cord syndrome	G822	Paraplegia unspecified
Acute nerve root/cord syndrome	G823	Flaccid tetraplegia
Acute nerve root/cord syndrome	G824	Spastic tetraplegia
Acute nerve root/cord syndrome	G825	Tetraplegia unspecified
Acute nerve root/cord syndrome	G952	Cord compression unspecified
Acute nerve root/cord syndrome	G992	Myelopathy in diseases classified elsewhere
Subarachnoid haemorrhage	I600	Subarachnoid haemorrhage from carotid siphon and bifurcation
Subarachnoid haemorrhage	I601	Subarachnoid haemorrhage from middle cerebral artery

For peer review only

Subarachnoid haemorrhage	I602	Subarachnoid haemorrhage from anterior communicating artery
Subarachnoid haemorrhage	I603	Subarachnoid haemorrhage from posterior communicating artery
Subarachnoid haemorrhage	I604	Subarachnoid haemorrhage from basilar artery
Subarachnoid haemorrhage	I605	Subarachnoid haemorrhage from vertebral artery
Subarachnoid haemorrhage	I606	Subarachnoid haemorrhage from other intracranial arteries
Subarachnoid haemorrhage	I607	Subarachnoid haemorrhage from intracranial artery, unspecified
Subarachnoid haemorrhage	I608	Other subarachnoid haemorrhage
Subarachnoid haemorrhage	I609	Subarachnoid haemorrhage, unspecified
Subarachnoid haemorrhage	I690	Sequelae of subarachnoid haemorrhage
Acute nerve root/cord syndrome	M470	Anterior spinal and vertebral artery compression syndromes
Acute nerve root/cord syndrome	M471	Other spondylosis with myelopathy
Acute nerve root/cord syndrome	M490	Tuberculosis of spine
Acute nerve root/cord syndrome	M500	Cervical disc disorder with myelopathy - linked to G992A
Acute nerve root/cord syndrome	M501	Cervical disc disorder with radiculopathy
Acute nerve root/cord syndrome	M510	Lumbar and other intervertebral disc disorders with myelopathy
Acute nerve root/cord syndrome	M511	M51.1(dagger) Lumbar and other intervertebral disc disorders with radiculopathy (G55.1(asterix))
Acute nerve root/cord syndrome	M612	Paralytic calcification and ossification of muscle (Myositis ossificans associated with quadriplegia or paraplegia)
Head Injuries	S040	Injury of optic nerve and pathways

For peer review only

Head Injuries	S041	Injury of oculomotor nerve
Head Injuries	S042	Injury of trochlear nerve
Head Injuries	S043	Injury of trigeminal nerve
Head Injuries	S044	Injury of abducent nerve
Head Injuries	S045	Injury of facial nerve
Head Injuries	S046	Injury of acoustic nerve
Head Injuries	S047	Injury of accessory nerve
Head Injuries	S048	Injury of other cranial nerves
Head Injuries	S049	Injury of unspecified cranial nerve
Head Injuries	S060	Concussion
Head Injuries	S061	Traumatic cerebral oedema
Head Injuries	S062	Diffuse brain injury
Head Injuries	S063	Focal brain injury
Head Injuries	S064	Epidural haemorrhage
Head Injuries	S065	Traumatic subdural haemorrhage
Subarachnoid haemorrhage	S066	Traumatic subarachnoid haemorrhage
Head Injuries	S067	Intracranial injury with prolonged coma
Head Injuries	S068	Other intracranial injuries
Head Injuries	S069	Intracranial injury unspecified
Acute nerve root/cord syndrome	T093	Injury of spinal cord level unspecified

For peer review only

The RECORD statement – checklist of items, extended from the STROBE statement, that should be reported in observational studies using routinely collected health data.

	Item No.	STROBE items	Location in manuscript where items are reported	RECORD items	Location in manuscript where items are reported
**Title and abstract**					
	1	(a) Indicate the study's design with a commonly used term in the title or the abstract (b) Provide in the abstract an informative and balanced summary of what was done and what was found	Abstract, p2	RECORD 1.1: The type of data used should be specified in the title or abstract. When possible, the name of the databases used should be included.<br><br>RECORD 1.2: If applicable, the geographic region and timeframe within which the study took place should be reported in the title or abstract.<br><br>RECORD 1.3: If linkage between databases was conducted for the study, this should be clearly stated in the title or abstract.	Abstract, p2<br><br>Abstract, p2<br><br>Not applicable
**Introduction**					
Background rationale	2	Explain the scientific background and rationale for the investigation being reported	Introduction, p3		
Objectives	3	State specific objectives, including any prespecified hypotheses	Introduction, p3		
**Methods**					
Study Design	4	Present key elements of study design early in the paper	Methods, pp4-5		
Setting	5	Describe the setting, locations, and relevant dates, including periods of recruitment, exposure, follow-up, and data collection	Methods, pp4-5		

For peer review only

Participants	6	*(a) Cohort study* - Give the eligibility criteria, and the sources and methods of selection of participants. Describe methods of follow-up<br>*Case-control study* - Give the eligibility criteria, and the sources and methods of case ascertainment and control selection. Give the rationale for the choice of cases and controls<br>*Cross-sectional study* - Give the eligibility criteria, and the sources and methods of selection of participants<br><br>*(b) Cohort study* - For matched studies, give matching criteria and number of exposed and unexposed<br>*Case-control study* - For matched studies, give matching criteria and the number of controls per case	Retrospective empirical research based on routine healthcare data	RECORD 6.1: The methods of study population selection (such as codes or algorithms used to identify subjects) should be listed in detail. If this is not possible, an explanation should be provided.<br><br>RECORD 6.2: Any validation studies of the codes or algorithms used to select the population should be referenced. If validation was conducted for this study and not published elsewhere, detailed methods and results should be provided.<br><br>RECORD 6.3: If the study involved linkage of databases, consider use of a flow diagram or other graphical display to demonstrate the data linkage process, including the number of individuals with linked data at each stage.	Methods, pp4-5<br><br>Methods, pp4-5<br><br>Not applicable
Variables	7	Clearly define all outcomes, exposures, predictors, potential confounders, and effect modifiers. Give diagnostic criteria, if applicable.	Methods, pp4-5 and appendix 1	RECORD 7.1: A complete list of codes and algorithms used to classify exposures, outcomes, confounders, and effect modifiers should be provided. If these cannot be reported, an explanation should be provided.	Appendix 1
Data sources/ measurement	8	For each variable of interest, give sources of data and details of methods of assessment (measurement).<br>Describe comparability of assessment methods if there is more than one group	Methods, pp4-5		

For peer review only

Bias	9	Describe any efforts to address potential sources of bias	Methods, pp4-5		
Study size	10	Explain how the study size was arrived at	Methods, pp4-5		
Quantitative variables	11	Explain how quantitative variables were handled in the analyses. If applicable, describe which groupings were chosen, and why	Methods, pp4-5		
Statistical methods	12	(a) Describe all statistical methods, including those used to control for confounding<br>(b) Describe any methods used to examine subgroups and interactions<br>(c) Explain how missing data were addressed<br>(d) *Cohort study* - If applicable, explain how loss to follow-up was addressed<br>*Case-control study* - If applicable, explain how matching of cases and controls was addressed<br>*Cross-sectional study* - If applicable, describe analytical methods taking account of sampling strategy<br>(e) Describe any sensitivity analyses	Methods, pp4-5		
Data access and cleaning methods		..		RECORD 12.1: Authors should describe the extent to which the investigators had access to the database population used to create the study population.	Methods, pp4-5<br><br>Methods, pp4-5

For peer review only

				RECORD 12.2: Authors should provide information on the data cleaning methods used in the study.	
Linkage		..		RECORD 12.3: State whether the study included person-level, institutional-level, or other data linkage across two or more databases. The methods of linkage and methods of linkage quality evaluation should be provided.	Not applicable
**Results**					
Participants	13	(a) Report the numbers of individuals at each stage of the study (*e.g.*, numbers potentially eligible, examined for eligibility, confirmed eligible, included in the study, completing follow-up, and analysed)<br>(b) Give reasons for non-participation at each stage.<br>(c) Consider use of a flow diagram	Results, pp6-9	RECORD 13.1: Describe in detail the selection of the persons included in the study (*i.e.,* study population selection) including filtering based on data quality, data availability and linkage. The selection of included persons can be described in the text and/or by means of the study flow diagram.	Results, pp6-9
Descriptive data	14	(a) Give characteristics of study participants (*e.g.*, demographic, clinical, social) and information on exposures and potential confounders<br>(b) Indicate the number of participants with missing data for each variable of interest<br>(c) *Cohort study* - summarise follow-up time (*e.g.*, average and total amount)	Results, pp6-9		
Outcome data	15	*Cohort study* - Report numbers of outcome events or summary measures over time<br>*Case-control study* - Report numbers in each exposure	Results, pp6-9		

		category, or summary measures of exposure<br>*Cross-sectional study* - Report numbers of outcome events or summary measures			
Main results	16	(a) Give unadjusted estimates and, if applicable, confounder-adjusted estimates and their precision (e.g., 95% confidence interval). Make clear which confounders were adjusted for and why they were included<br>(b) Report category boundaries when continuous variables were categorized<br>(c) If relevant, consider translating estimates of relative risk into absolute risk for a meaningful time period	Results, pp6-9		
Other analyses	17	Report other analyses done—e.g., analyses of subgroups and interactions, and sensitivity analyses	Results, pp6-9		
**Discussion**					
Key results	18	Summarise key results with reference to study objectives	Discussion, pp10-11		
Limitations	19	Discuss limitations of the study, taking into account sources of potential bias or imprecision. Discuss both direction and magnitude of any potential bias	Discussion, pp10-11	RECORD 19.1: Discuss the implications of using data that were not created or collected to answer the specific research question(s). Include discussion of misclassification bias, unmeasured confounding, missing data, and changing eligibility over time, as they pertain to the study being reported.	Discussion, pp10-11
Interpretation	20	Give a cautious overall interpretation of results considering objectives,	Discussion, pp10-11		

For peer review only

		limitations, multiplicity of analyses, results from similar studies, and other relevant evidence			
Generalisability	21	Discuss the generalisability (external validity) of the study results	Discussion, pp10-11		
**Other Information**					
Funding	22	Give the source of funding and the role of the funders for the present study and, if applicable, for the original study on which the present article is based	No study-specific funding		
Accessibility of protocol, raw data, and programming code		..	Methods, pp4-5 and appendix 1	RECORD 22.1: Authors should provide information on how to access any supplemental information such as the study protocol, raw data, or programming code.	Corresponding author details

*Reference: Benchimol EI, Smeeth L, Guttmann A, Harron K, Moher D, Petersen I, Sørensen HT, von Elm E, Langan SM, the RECORD Working Committee. The REporting of studies Conducted using Observational Routinely-collected health Data (RECORD) Statement. *PLoS Medicine* 2015; in press.

*Checklist is protected under Creative Commons Attribution (CC BY) license.

For peer review only

BMJ Open

Numbers and types of neurological emergencies in England and the influence of socio-economic deprivation: a retrospective analysis of Hospital Episode Statistics data

Journal:	*BMJ Open*
Manuscript ID	bmjopen-2022-061843.R2
Article Type:	Original research
Date Submitted by the Author:	13-Sep-2022
Complete List of Authors:	Jackson, Michael; United Kingdom Department of Health and Social Care, Office for Health Improvement and Disparities (OHID)<br>Szczepaniak, Marta; United Kingdom Department of Health and Social Care, Office for Health Improvement and Disparities (OHID)<br>Wall, Jasmine; Lancaster University; Lancashire Teaching Hospitals NHS Foundation Trust<br>Maskery, Mark; Lancaster University; Lancashire Teaching Hospitals NHS Foundation Trust<br>Mummery, Catherine; University College London Hospitals NHS Foundation Trust<br>Morrish, Paul; Great Western Hospital Foundation NHS Trust<br>Williams, Adrian; University Hospitals Birmingham NHS Foundation Trust; NHS England<br>Knight, Joanne; Lancaster University<br>Emsley, Hedley; Lancaster University; Lancashire Teaching Hospitals NHS Foundation Trust
**Primary Subject Heading**:	Neurology
Secondary Subject Heading:	Epidemiology
Keywords:	Epilepsy < NEUROLOGY, Neurology < INTERNAL MEDICINE, Public health < INFECTIOUS DISEASES

SCHOLARONE™
Manuscripts

I, the Submitting Author has the right to grant and does grant on behalf of all authors of the Work (as defined in the below author licence), an exclusive licence and/or a non-exclusive licence for contributions from authors who are: i) UK Crown employees; ii) where BMJ has agreed a CC-BY licence shall apply, and/or iii) in accordance with the terms applicable for US Federal Government officers or employees acting as part of their official duties; on a worldwide, perpetual, irrevocable, royalty-free basis to BMJ Publishing Group Ltd ("BMJ") its licensees and where the relevant Journal is co-owned by BMJ to the co-owners of the Journal, to publish the Work in this journal and any other BMJ products and to exploit all rights, as set out in our licence.

The Submitting Author accepts and understands that any supply made under these terms is made by BMJ to the Submitting Author unless you are acting as an employee on behalf of your employer or a postgraduate student of an affiliated institution which is paying any applicable article publishing charge ("APC") for Open Access articles. Where the Submitting Author wishes to make the Work available on an Open Access basis (and intends to pay the relevant APC), the terms of reuse of such Open Access shall be governed by a Creative Commons licence – details of these licences and which Creative Commons licence will apply to this Work are set out in our licence referred to above.

Other than as permitted in any relevant BMJ Author's Self Archiving Policies, I confirm this Work has not been accepted for publication elsewhere, is not being considered for publication elsewhere and does not duplicate material already published. I confirm all authors consent to publication of this Work and authorise the granting of this licence.

For peer review only

Numbers and types of neurological emergencies in England and the influence of socio-economic deprivation: a retrospective analysis of Hospital Episode Statistics data

A retrospective empirical research study

Jackson M[1], Szczepaniak M[1], Wall J[2,3], Maskery M[2,3], Mummery C[4], Morrish P[5], Williams A[6,7], Knight J[1,2], Emsley HCA[1,2,3]

[1]Neurology & Dementia Intelligence team part of Public Health Analysis Group, Office for Health Improvement and Disparities (OHID), Department of Health and Social Care, UK (Formerly Public Health England); [2]Lancaster Medical School, Lancaster University, Lancaster, UK; [3]Lancashire Teaching Hospitals NHS Foundation Trust, Preston, UK; [4]Dementia Research Centre, National Hospital for Neurology and Neurosurgery, University College London Hospitals NHS Foundation Trust, London, UK; [5]Greater Western Hospitals NHS Foundation Trust, Swindon, UK; [6]University Hospitals Birmingham NHS Foundation Trust, Birmingham, UK; [7]Neuroscience Clinical Reference Group, NHS England, London, UK

Author email addresses

Michael Jackson	michael.jackson@dhsc.gov.uk
Marta Szczepaniak	marta.szczepaniak@dhsc.gov.uk
Jasmine Wall	jasmine.wall@lthtr.nhs.uk
Mark Maskery	mark.maskery@lthtr.nhs.uk
Cath Mummery	cath.mummery@nhs.net
Paul Morrish	p.morrish@nhs.net
Adrian Williams	adrian.williams@uhb.nhs.uk
Jo Knight	jo.knight@lancaster.ac.uk
Hedley Emsley*	hedley.emsley@lancaster.ac.uk

*corresponding author address. Department of Neurology, Royal Preston Hospital, Lancashire Teaching Hospitals NHS Foundation Trust, Sharoe Green Lane, Preston, PR2 9HT

For peer review only

Abstract

Objectives In this first large-scale analysis of neurological emergency admissions in England, we determine the number and types of emergency admissions with neurological emergency diagnostic codes, how many are under the care of a neurologist or neurosurgeon, and how such admissions vary by levels of deprivation

Design Retrospective empirical research employing a derived list of neurological emergency diagnostic codes

Setting This study used the Hospital Episode Statistics (HES) dataset for the financial year 2019/2020 based on 17 million in-year inpatient admissions in England including 6.5 million (100%) emergency admissions with any diagnosis codes

Results There were 1.4 million (21.2%) emergency inpatient admissions with a mention of any neurological code, approx. 248,455 (3.8%) with mention of a specific neurological emergency code from the derived list, and 72,485 (1.1%) included such a code as the primary reason for admission. The highest number of in-year admissions for adults was for epilepsy (145,995), with epilepsy as the primary diagnostic code in 15,945 (10.9%). Acute nerve root/spinal cord syndrome (41,215), head injury (29,235) and subarachnoid haemorrhage (18,505) accounted for the next three highest number of admissions. 3,230 (1.4%) in-year emergency hospital admissions with mention of a neurological emergency code were under the care of a neurologist or neurosurgeon, with only 1,315 (0.9%) admissions with mention of an epilepsy code under a neurologist. There was significant variation for epilepsy and functional neurological disorders (FNDs) in particular by Index of Multiple Deprivation decile. The association between deprivation and epilepsy and FND was significant with p-values of 2.5e-6, 1.5e-8 respectively.

Conclusions This study has identified important findings in relation to the burden of neurological emergency admissions but further work is needed, with greater clinical engagement in diagnostic coding, to better understand the implications for workforce and changes to service delivery needing to be implemented.

For peer review only

Strengths and limitations

- First analysis of its type arising from an academic partnership with the Office for Health Improvement and Disparities, utilising Hospital Episode Statistics data
- Large-scale analysis based on 6.5 million emergency admissions increases robustness of findings
- Data used was collected for the purposes of healthcare delivery rather than research
- Retrospective nature of study limits interpretation of some findings such as the nature of suspected cauda equina presentations
- Inclusion of emergency admissions with mention of neurological condition rather than solely neurological emergencies *per se* on account of coding accuracy concerns

For peer review only

Introduction

Medical emergency admissions continue to rise across the UK. They are more than twice as likely among the most deprived population decile by comparison with mean deprivation, reinforcing the point that challenges are social as well as medical[1,2]. Many emergency admissions to hospital are considered to be avoidable and patients often stay in hospital longer than is necessary[3]. Emergency admissions are also disruptive of elective care delivery, prolonging waiting times. Hospital Episodes Statistics (HES) data for 2016/17 suggest that approximately 18% of medical emergency admissions included a mention of neurological conditions including stroke and dementia, totalling 1.09 million, a rise of 21% over the previous 5 years[4]. Excess mortality has also been reported among patients with epilepsy[5]. At a time when costs are rising and resources are increasingly under pressure, it is all the more important to have a better understanding of neurological emergency admissions and underlying factors such as social deprivation.

There has been little by way of systematic evaluation of data pertaining to neurological emergency admissions. Previous work has used a much broader definition, counting neurological conditions present in patients admitted as an emergency, including stroke and dementia, rather than neurological emergencies *per se*. The present work has by necessity involved the creation of a list defining neurological emergencies. Much remains unknown about neurological emergency admissions, including their total number, the distribution of neurological conditions, and the effect of socio-economic deprivation. There have been some studies investigating the influence of comorbidities and race in the United States [6,7]. This study represents the first large scale analysis of neurological emergency admissions in England, and the first OHID-academic partnership in neurology. For the first time, the data provide some insight into the number and types of emergency admissions with neurological emergency codes, the number of such admissions under the care of a neurologist, and how such admissions vary on the basis of factors such as levels of deprivation.

For peer review only

Methods

Defining neurological emergencies

For the purposes of this work it was necessary to create a list of conditions typically regarded as neurological emergencies, and to map these to the relevant ICD-10 codes. An initial set of neurological conditions was proposed by AW as the basis for the study, comprising causes of acute paralysis such as Guillain-Barré syndrome, myasthenic crisis, encephalitis, spinal cord compression and status epilepticus. The ICD-10 codes most closely matching these 'index' entities were compiled into a list. Other ICD-10 codes in close hierarchical proximity to the 'index' ICD-10 codes were added from a previously published Public Health England (PHE) neurology code collection [8], which comprised 581 4-digit ICD-10 codes for 19 condition groups. The 'long list' of candidate neurological emergency codes, consisting of 136 ICD-10 codes, was reviewed iteratively by the project team and clinicians working alongside, and refined into a short list of 96 4-digit ICD-10 codes (appendix 1) for use in the work reported here, subsequently referred to as the 'derived list' [of neurological emergency codes]. The selection process used clinical judgement to decide which conditions or groups of conditions to include or exclude, with reference to entities classically considered to be neurological emergencies. Of particular note, stroke, despite being a classical neurological emergency, was excluded from this exercise given that stroke is already the subject of the existing national audit system (SSNAP). Codes for conditions presenting with secondary headache have been included; codes for primary headache disorders have not been included in the present work. Also of note was the decision to include functional neurological disorders (FNDs) on the basis that we believed these to contribute significantly to the total number of emergency hospital admissions for neurological conditions, as well as the need for patients with FND to be referred to specialists with expertise in neurological diagnosis [9]. FNDs is a term given to neurological features that are not due to any physical neurological disorder. We have defined relevant terminology in Box 1.

Defining study group

The hospital activity included in this study were admissions to hospital occurring during the financial year 2019/2020 in England – described as an in-year admission, where the admission was classed as an emergency (non-elective) admission to hospital and where the selected neurological emergency codes are in any diagnosis position, on any consultant episode during the hospital stay.

Data source, data extraction and statistics

The data source used in this study is the Admitted Patient Care version of the Hospital Episode Statistics dataset for the financial year 2019/2020. Data extraction was undertaken by the OHID Neurology Dementia Intelligence team using Structured Query Language (SQL) with further data manipulation, including descriptive statistics, using Microsoft Excel. Counts and percentages were extracted and/or calculated for various categories, confidence intervals were estimated and data was visualised in a range of chart types. Other than for the data in table 1 and the associated summary text, the paediatric population was excluded from further analyses. Chi squared tests were used to determine if the distribution of admission by primary code versus secondary was independent of diagnosis and to assess the independence of diagnosis with involvement of a neurologist at admission, later in the spell or never. A Cramérs V was used to assess independence of admission with indices of deprivation; this was stratified by a range of diagnosis type. For the first two analyses a code assigned to admission is the unit of analysis, for the third analysis the unit of analysis is admission of an individual.

For peer review only - http://bmjopen.bmj.com/site/about/guidelines.xhtml

Patient and public involvement

This was a retrospective empirical research study based on a secondary analysis of routinely collected anonymised clinical data. Patient and public involvement was not applicable.

Box 1. Relevant terminology

People admitted to hospital will always have a responsible medical officer (consultant) assigned for the time under that consultant, or episode (**consultant episode**).

The consultant episode at the start of the hospital stay is the **admission episode**; the episode at the end of the hospital spell is called the **discharge episode**.

Hospital admissions are the start of **hospital spells**, which comprise one or more consultant episodes.

ICD-10 – International Statistical Classification of Diseases and Related Health Problems (ICD) (10th edition) codes, or **hospital diagnosis codes**. ICD-10 is a legally mandated health data standard.

Conditions present during the consultant episode can be described using up to 20 corresponding ICD-10 codes . Hospital stays with multiple consultant episodes can have very differing collections of ICD-10 codes recorded on each episode.

The ICD-10 code that is recorded in the first position on a consultant episode is known as the **primary diagnosis**. Subsequent codes on the same episodes are considered **secondary diagnoses** and may represent conditions of lesser importance for this particular consultant episode or other long-term conditions/co-morbidities of the patient.

Hospital episode statistics (HES) are financial year based. Stays in hospital can cross the financial year timelines, therefore the number of admissions and discharges in any one financial year may not be equal. **In-year hospital admissions** relates to the count of all hospital stays that start in the financial year. **In-year hospital discharges** relate to the count of all hospital stays that end during the financial year. **HES ID** is a pseudonymised unique identifier for each individual patient, which can be used to group hospital episodes and spells by individual

Results

Emergency inpatient admissions with any neurological codes or neurological emergency codes

Table 1 shows totals for inpatient activity for all ages by admission type for 2019/2020. Of a total of over 17 million inpatient admissions, 6.5 million were emergency admissions. Among these, 1.4 million (21%) were emergency inpatient admissions with a mention of any neurological code (including dementia and stroke). Emergency inpatient admissions with a specific neurological emergency code from the derived list (appendix 1) represented 3.8% of all emergency inpatient admissions, and such a code was the primary reason for admission in 1.1% of all emergency inpatient admissions. Among all emergency adult in-year admissions by spell with specific neurological diagnosis codes from the derived list 132,485 (54.4%) comprised a single episode.

The number of unique adult patients (using HESID as a proxy) with in-year admissions by spell with specific neurological diagnosis codes from the derived list was 167,735. 75.6% (126,800) of these individuals have only one admission.

Table 1: Inpatient hospital activity by admission type; all ages; England; 2019/20

	Counts	Percentage of all emergency inpatient admissions
All inpatient admissions: in-year admissions by spell with any diagnosis codes	17,183,220	NA
Emergency inpatient admissions: in-year admissions by spell with any diagnosis codes	6,506,970	100%
Emergency inpatient admissions with a mention of any neurological code (including dementia and stroke): in-year admissions by spell	1,380,975	21.2%
Emergency inpatient admissions with neurological emergency codes: in-year admissions by spell with specific neurological diagnosis codes	248,455	3.8%
Emergency inpatient admissions with neurological emergency code as primary reason for admission: in-year admissions by spell with specific neurological diagnosis codes	72,485	1.1%

Source: OHID Neurology Dementia Intelligence using Hospital Episode Statistics Admited Patient Care, NHS Digital

For peer review only

Neurological conditions

Table 2 shows adult emergency inpatient hospital admissions with a mention of neurological emergency codes. Among a total of 237,755 such admissions, 65,060 (27.4%) had a neurological emergency code as a primary diagnostic code on the starting episode. Similar percentages were noted for acute paralysis (24.5%), functional disorders (29.8%), acute nerve root/cord syndrome (30.7%) and encephalitis (34.2%).

However we see a strikingly different pattern for epilepsy, subarachnoid haemorrhage (SAH) and head injury (HI). The highest number of in-year admissions was for epilepsy (145,995), yet the percentage of those with epilepsy as the primary diagnostic code was only 10.9%. By comparison, the primary diagnostic codes for head injury appeared in 73.1% of 29,235 admissions for this condition, and similarly for subarachnoid haemorrhage the primary diagnostic code appeared in 57.9% of 18,505 admissions. The p-value of the association of diagnosis with primary code is $<10^{-6}$.

Table 2: Emergency inpatient hospital admissions with a mention of neurological emergency codes; adults aged 18 years and over; England; 2019/20

	In-year admissions	In-year admissions with primary diagnosis on starting episode	Primary diagnosis as % of in-year admissions
neurological emergencies	237,755	65,060	27.4%
emergency acute nerve root/cord syndrome	41,215	12,655	30.7%
emergency acute paralysis	2,600	635	24.5%
emergency encephalitis	5,240	1,790	34.2%
emergency epilepsy	145,995	15,945	10.9%
emergency functional disorders	6,545	1,950	29.8%
emergency head injuries	29,235	21,365	73.1%
emergency subarachnoid haemorrhage	18,505	10,720	57.9%

Source: OHID Neurology Dementia Intelligence using Hospital Episode Statistics Admited Patient Care, NHS Digital

For peer review only

Proportion of admissions under a neurologist

Figure 1 shows the percentage of in-year adult emergency hospital admissions with mention of a neurological emergency code under the care of a consultant in neurology or neurosurgery. Overall, among 237,755 such admissions, 3,230 (1.4%) were under the care of a consultant neurologist and 10,760 (4.5%) were under the care of a consultant neurosurgeon, with the majority of the admissions under a neurosurgeon having SAH or HI diagnostic codes (see supplementary table 1). Only 1,315 (0.9%) of admissions with mention of an epilepsy diagnostic code were under the care of a consultant neurologist. The p-value of the association of diagnosis with care under a neurologist either at admission/later in the spell or not at all is $<10^{-6}$.

Influence of socio-economic deprivation

Figure 2 shows a proxy for the number of people admitted (total, functional, epilepsy, SAH) by deciles of deprivation using the Indices of Multiple Deprivation [10] (see also supplementary table 2). This shows that the number of people admitted varies significantly by social deprivation. This pattern is seen for the total admissions with mention of neurological emergency codes, but is more apparent for epilepsy and is most striking for functional neurological disorders, where the greatest number were in the most deprived and the fewest in the least deprived (p-values 1.6e-4, 2.5e-6, 1.5e-8 respectively). By comparison, admissions with mention of SAH did not exhibit much variation by social deprivation (p-value=0.01).

For peer review only

Discussion

In this first large-scale analysis of 1.4 million emergency inpatient admissions in England for 2019/2020 with a mention of any neurological code, there were 248,455 with mention of a specific neurological emergency code from the derived list. The highest number was for epilepsy (145,995) with the next three most frequent causes being acute nerve root/spinal cord syndrome, head injury and subarachnoid haemorrhage. 1.4% of admissions with mention of a neurological emergency code were under a neurologist or neurosurgeon, with only 0.9% of those with mention of an epilepsy code under a neurologist. The greatest number of admissions were in the most deprived, particularly for epilepsy and functional neurological disorders. Being able to determine the proportion of emergency admissions that constitute neurological emergency presentations is clearly crucial for service and clinical pathway design and workforce planning.

It was necessary to formulate a list of neurological emergencies and associated list of ICD-10 codes as a prelude to the analyses in this study. ICD-10 codes are designed and assigned with a focus on healthcare delivery rather than research. We took a pragmatic approach based on current UK practice, taking into account factors such as existing well defined pathways of care (eg acute stroke) or the absence of well defined pathways (eg FND). Although perhaps not classically considered neurological emergencies, FND presentations are increasingly common. This is with respect to their impact both the individual and upon healthcare resources, resources which are often used inappropriately and not in alignment with the needs of such patients. The approach taken to derive the list of neurological emergencies, as well as the approach to analysis of the data, took into account the nature of the dataset being used.

We found that among adult emergency admissions with mention of a neurological emergency code, this was the primary diagnostic code in 27.4% overall. However, despite epilepsy accounting for the greatest number of admissions in our dataset, epilepsy appeared as the primary diagnostic code in only 10.9% by comparison with 73.1% for head injury 57.9% for SAH. It is important to note that epilepsy codes have been included among the neurological emergency codes but may reflect epilepsy as a comorbidity and not the reason for emergency admission, hence the comparatively low proportion of patients with epilepsy codes as the primary diagnostic code. A proportion of patients assigned epilepsy codes may have had dissociative seizures (DS). Admissions with mention of epilepsy codes merit further analysis in future work, particularly given recent data on epilepsy related mortality [5]. The current analysis cannot explain the differences between conditions in the proportion of relevant primary diagnostic codes being assigned. However, it is conceivable that the high proportion of head injury admissions with head injury as the primary diagnostic code, and similarly for SAH, may reflect the relative ease and pace of establishing the primary reason for admission.

We found that among emergency admissions with mention of a neurological emergency code, 1.4% were under the care of a consultant neurologist and 4.5% were under the care of a consultant neurosurgeon; only 0.9% with mention of an epilepsy diagnostic code were under the care of a consultant neurologist. Many admissions with single episodes not under neurology or neurosurgery also suggests many patients may not receive input from these disciplines despite having neurological codes assigned. Although the percentage of admissions under a consultant neurologist may appear disproportionately low given the greater number of neurologists by comparison with neurosurgeons, this may partly reflect the different balance of commitments between outpatient and inpatient pathways between neurology and neurosurgery. These findings are in keeping with previous data suggesting that a low proportion of epilepsy emergency admissions are under a neurologist. Further work, such as the prospective capture of neurological emergency codes with greater clinical

For peer review only

engagement to improve their accuracy, is needed, but our findings are in general agreement with the recognised urgent need to address workforce capacity to support neurological emergency admissions.

The present study highlights additional concerns, particularly the apparent relationship between admissions for FND and socioeconomic deprivation. We know that in many areas in England there is a significant gap in terms of provision of appropriate services for patients with FND [11], very likely to increase the likelihood of re-presentation as an emergency. A better understanding of emergency admissions for FND is needed to determine how clinical pathways can be improved for such patients. The retrospective nature of the present study limits interpretation of findings such as acute nerve root/spinal cord syndrome being arguably more frequent than expected; one possibility is a rise in 'scan-negative' cauda equina syndrome, itself another potential FND[9].

An important limitation of the methodology employed by this study is the inclusion of emergency admissions which mention neurological conditions, ie admissions which include codes deemed, as part of this study, relevant for neurological emergencies. These codes could appear at any position in the patient's diagnostic coding, and during any episode(s) comprising the given spell; implicit in this approach is the possibility that the neurological condition was not the reason for the emergency admission. This, of course, is different to the inclusion of neurological emergencies *per se*, where the primary cause of the emergency admission was neurological. Previous work has raised concern about the accuracy of current data collection methods, including the erroneous assignment of neurological codes [12].

Conclusion We have for the first time quantified the number of neurological emergency admissions across England, the distribution of conditions, the proportion under the care of a neurologist or neurosurgeon and evidence of variation by social deprivation. Further work, including improved clinical engagement with coding to ensure data accuracy, is required to understand the drivers of these findings. Further analyses are also needed to investigate issues such as the effect of hospital setting (eg district general hospital vs neurosciences centre), the point in the spell at which the neurological diagnosis is made and the influence of neurologist involvement. This includes neurological emergency admissions for FND. These activities will inform clinical pathway change across primary and secondary care to reduce avoidable admissions and improve care.

For peer review only

Contributorship statement

MJ, MS, JK and HE conceived and designed the work and jointly undertook the analysis and interpretation of the data. MJ, JK and HE jointly drafted the manuscript and revised it critically for important intellectual content. AW contributed to the conception of the work. JW, MM, CM, PM and AW contributed to the development of the derived list of neurological emergency diagnostic codes. All authors approved the final version.

The corresponding author attests that all listed authors meet authorship criteria and that no others meeting the criteria have been omitted.

Competing interests

The authors have no relevant competing interests to declare.

Funding

This study received no specific funding.

Data availability statement

Information relating to access to HES data used for this study can be found at https://digital.nhs.uk/data-and-information/data-tools-and-services/data-services/hospital-episode-statistics/users-uses-and-access-to-hospital-episode-statistics.

Ethics statement

Ethics Committee approval was not required as the research presented in this manuscript comprises a secondary analysis of routinely collected anonymised clinical data.

For peer review only

References

1. McCormick B, Hill P-S, Poteliakhoff E. 2012. Are hospital services used differently in deprived areas? Evidence to identify commissioning challenges. http://chseo.org.uk/downloads/wp2-hospitalservices-deprivedareas.pdf (accessed December 2021)
2. Purdy S. 2010. Avoiding hospital admissions. London: The King's Fund. https://www.kingsfund.org.uk/sites/default/files/Avoiding-Hospital-Admissions-Sarah-Purdy-December2010.pdf (accessed January 2022)
3. National Audit Office. 2013. Emergency admissions to hospital: managing the demand. https://www.nao.org.uk/report/emergency-admissions-hospitals-managing-demand/ (accessed December 2021)
4. The Neurological Alliance. 2019. Neuro Numbers 2019. https://www.neural.org.uk/assets/pdfs/neuro-numbers-2019.pdf (accessed December 2021)
5. Public Health England. 2018. Deaths associated with neurological conditions data briefing. https://www.gov.uk/government/publications/deaths-associated-with-neurological-conditions (accessed January 2022)
6. Sajatovic M, Welter E, Tatsuoka C, Perzynski AT, Einstadter D. Electronic medical record analysis of emergency room visits and hospitalizations in individuals with epilepsy and mental illness comorbidity. Epilepsy Behav. 2015 Sep;50:55-60. doi: 10.1016/j.yebeh.2015.05.016. Epub 2015 Jun 25. PMID: 26119621; PMCID: PMC4793963.
7. Fantaneanu TA, Hurwitz S, van Meurs K, Llewellyn N, O'Laughlin KN, Dworetzky BA. Racial differences in Emergency Department visits for seizures. Seizure. 2016 Aug;40:52-6. doi: 10.1016/j.seizure.2016.06.007. Epub 2016 Jun 16. PMID: 27344498.
8. Public Health England. 2021. Neurology and Dementia Intelligence: ICD10 code collections. https://fingertips.phe.org.uk/profile-group/mental-health/supporting-information/ndi-code-collections (accessed December 2021)
9. Bennett K, Diamond C, Hoeritzauer I, Gardiner P, McWhirter L, Carson A, Stone J. A practical review of functional neurological disorder (FND) for the general physician. Clin Med (Lond). 2021 Jan;21(1):28-36. doi: 10.7861/clinmed.2020-0987. PMID: 33479065; PMCID: PMC7850207.
10. Ministry of Housing, Communities & Local Government. 2019. English indices of deprivation 2019. https://www.gov.uk/government/statistics/english-indices-of-deprivation-2019 (accessed December 2021)
11. O'Keeffe S, Chowdhury I, Sinanaj A, Ewang I, Blain C, Teodoro T, Edwards M, Yogarajah M. A Service Evaluation of the Experiences of Patients With Functional Neurological Disorders Within the NHS. Front Neurol. 2021 May 31;12:656466. doi: 10.3389/fneur.2021.656466. PMID: 34135848; PMCID: PMC8200476.
12. Kanagaratnam M, Boodhoo A, MacDonald BK, Nitkunan A. Prevalence of acute neurology: a 2-week snapshot in a district general hospital. Clin Med (Lond). 2020;20(2):169-173. doi:10.7861/clinmed.2019-0377

Figure Legends

Figure 1. Percentage of in-year emergency hospital admissions with mention of neurological emergency codes admitted under the care of a consultant neurologist or neurosurgeon; adults aged 18 years and over; England; 2019/20; source: OHID Neurology Dementia Intelligence using Hospital Episode Statistics Admitted Patient Care, NHS Digital.

[see separate figure 1 file]

Figure 2. Proportion of unique HESIDs (proxy for individuals) with an in-year neurological emergency hospital admission, by decile of deprivation and condition group; adults aged 18 years and over; Indices of Multiple Deprivation 2019; England; 2019/20; source: OHID Neurology Dementia Intelligence using Hospital Episode Statistics Admitted Patient Care, NHS Digital.

[see separate figure 2 file]

For peer review only

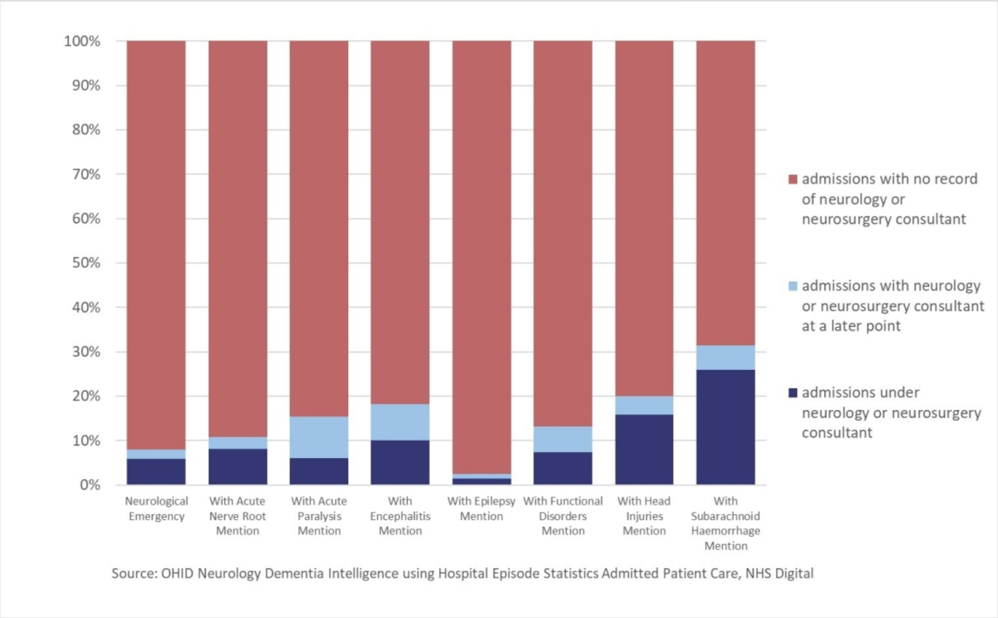

Figure 1. Percentage of in-year emergency hospital admissions with mention of neurological emergency codes admitted under the care of a consultant neurologist or neurosurgeon; adults aged 18 years and over; England; 2019/20; source: OHID Neurology Dementia Intelligence using Hospital Episode Statistics Admitted Patient Care, NHS Digital.

256x159mm (150 x 150 DPI)

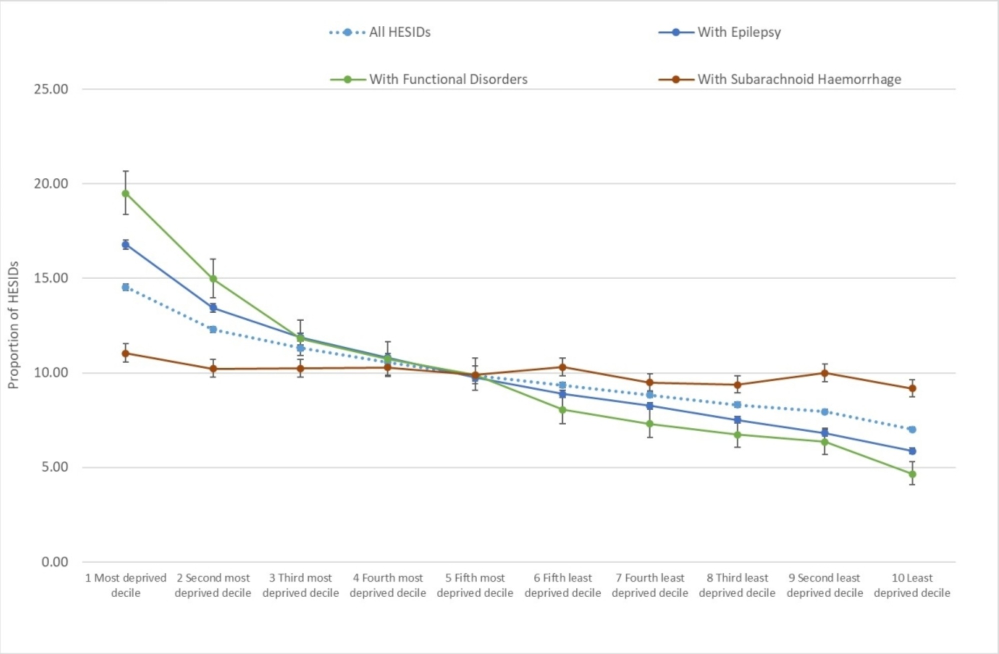

Figure 2. Proportion of unique HESIDs (proxy for individuals) with an in-year neurological emergency hospital admission, by decile of deprivation and condition group; adults aged 18 years and over; Indices of Multiple Deprivation 2019; England; 2019/20; source: OHID Neurology Dementia Intelligence using Hospital Episode Statistics Admitted Patient Care, NHS Digital.

258x168mm (150 x 150 DPI)

Supplementary Table 1: Percentage of in-year emergency hospital admissions with mention of neurological emergency codes admitted under the care of a consultant neurologist or neurosurgeon; adults aged 18 years and over; England; 2019/20

	Neurological Emergency	With Acute Nerve Root Mention	With Acute Paralysis Mention	With Encephalitis Mention	With Epilepsy Mention	With Functional Disorders Mention	With Head Injuries Mention	With Subarachnoid Haemorrhage Mention
admissions under neurology consultant	3,230	545	145	270	1,315	445	305	440
%	*1.36%*	*1.32%*	*5.58%*	*5.19%*	*0.90%*	*6.80%*	*1.04%*	*2.38%*
admissions under neurosurgery consultant	10,760	2,905	10	255	810	40	4,360	4,375
%	*4.52%*	*7.05%*	*0.42%*	*4.89%*	*0.56%*	*0.63%*	*14.91%*	*23.63%*
admissions under neurology or neurosurgery consultant	13,860	3,355	155	525	2,120	485	4,655	4,800
%	*5.83%*	*8.15%*	*6.00%*	*10.06%*	*1.45%*	*7.40%*	*15.92%*	*25.95%*
admissions with neurology consultant at a later point	3,150	435	250	410	1,355	370	390	320

For peer review only

%	*1.32%*	*1.06%*	*9.58%*	*7.81%*	*0.93%*	*5.67%*	*1.33%*	*1.72%*
admissions with neurosurgery consultant at a later point	2,265	795	*	55	165	10	945	835
%	*0.95%*	*1.93%*	*0.00%*	*1.07%*	*0.11%*	*0.17%*	*3.23%*	*4.52%*
admissions with neurology or neurosurgery consultant at a later point	5,000	1,110	245	430	1,465	375	1,200	1,010
%	*2.10%*	*2.70%*	*9.35%*	*8.21%*	*1.00%*	*5.72%*	*4.11%*	*5.49%*
admissions with no record of neurology or neurosurgery consultant	218,890	36,745	2,200	4,280	142,410	5,690	23,380	12,690
%	*92.07%*	*89.15%*	*84.64%*	*81.73%*	*97.54%*	*86.89%*	*79.97%*	*68.56%*
**in year admissions**	**237,755**	**41,215**	**2,600**	**5,240**	**145,995**	**6,545**	**29,235**	**18,505**
Source: OHID Neurology Dementia Intelligence using Hospital Episode Statistics Admited Patient Care, NHS Digital								

For peer review only

Supplementary Table 2

Deciles of Deprivation England		All HESIDs	With Epilepsy	With Functional Disorders	With Subarachnoid Haemorrhage
**1 Most deprived**	Count	23,600	14,970	910	1,770
	Percentage	14.5%	16.8%	19.5%	11.0%
**2**	Count	19,965	11,990	700	1,640
	Percentage	12.3%	13.4%	15.0%	10.2%
**3**	Count	18,365	10,595	555	1,640
	Percentage	11.3%	11.9%	11.8%	10.2%
**4**	Count	17,175	9,640	500	1,650
	Percentage	10.6%	10.8%	10.7%	10.3%
**5**	Count	16,005	8,700	465	1,590
	Percentage	9.9%	9.8%	9.9%	9.9%
**6**	Count	15,190	7,920	375	1,655
	Percentage	9.4%	8.9%	8.1%	10.3%
**7**	Count	14,340	7,365	340	1,525
	Percentage	8.8%	8.3%	7.3%	9.5%
**8**	Count	13,510	6,700	315	1,505
	Percentage	8.3%	7.5%	6.7%	9.4%
**9**	Count	12,920	6,080	300	1,605
	Percentage	8.0%	6.8%	6.3%	10.0%
**10 Least deprived**	Count	11,375	5,225	220	1,470
	Percentage	7.0%	5.9%	4.7%	9.2%

Source: OHID Neurology Dementia Intelligence using Hospital Episode Statistics Admitted Patient Care, NHS Digital

For peer review only

Appendix 1

Neurological Emergency ICD10 codes – 96 codes (short list)

NeuroEmergencyConditionGroup	NeuroEmergencyICD10CodeHES	ICD10_Level3Header
Acute paralysis	A521	Symptomatic neurosyphilis
Encephalitis	A830	Japanese encephalitis
Encephalitis	A831	Western equine encephalitis
Encephalitis	A832	Eastern equine encephalitis
Encephalitis	A833	St Louis encephalitis
Encephalitis	A834	Australian encephalitis
Encephalitis	A835	California encephalitis
Encephalitis	A836	Rocio virus disease
Encephalitis	A838	Other mosquito-borne viral encephalitis
Encephalitis	A839	Mosquito-borne viral encephalitis
Encephalitis	A840	Far Eastern tick-borne encephalitis [Russian spring-summer encephalitis]
Encephalitis	A841	Central European tick-borne encephalitis
Encephalitis	A848	Other tick-borne viral encephalitis
Encephalitis	A849	Tick-borne viral encephalitis
Encephalitis	A850	Enteroviral encephalitis
Encephalitis	A851	Adenoviral encephalitis
Encephalitis	A852	Arthropod-borne viral encephalitis, unspecified
Encephalitis	A858	Other specified viral encephalitis

Encephalitis	A86X	Unspecified viral encephalitis
Functional Disorders	F440	Dissociative amnesia
Functional Disorders	F441	Dissociative fugue
Functional Disorders	F442	Dissociative stupor
Functional Disorders	F443	Trance and possession disorders
Functional Disorders	F444	Dissociative motor disorders
Functional Disorders	F445	Dissociative convulsions
Functional Disorders	F446	Dissociative anaesthesia and sensory loss
Functional Disorders	F447	Mixed dissociative conversion disorders
Functional Disorders	F448	Other dissociative conversion disorders
Functional Disorders	F449	Dissociative conversion disorder unspecified
Encephalitis	G040	Acute disseminated encephalitis
Encephalitis	G041	Tropical spastic paraplegia
Encephalitis	G042	Bacterial meningoencephalitis and meningomyelitis NEC
Encephalitis	G048	Other encephalitis myelitis and encephalomyelitis
Encephalitis	G049	Encephalitis myelitis and encephalomyelitis unspecified
Encephalitis	G050	Encephalitis myelitis and encephalomyelitis in bacterial diseases classified elsewhere
Encephalitis	G051	Encephalitis myelitis and encephalomyelitis in viral diseases classified elsewhere
Encephalitis	G052	Encephalitis myelitis and encephalomyelitis in other infectious and parasitic diseases classified elsewhere
Encephalitis	G058	Encephalitis myelitis and encephalomyelitis in other diseases classified elsewhere
Acute nerve root/cord syndrome	G114	Hereditary spastic paraplegia
Epilepsy	G409	Epilepsy unspecified
Epilepsy	G410	Grand mal status epilepticus
Epilepsy	G411	Petit mal status epilepticus

For peer review only

Epilepsy	G412	Complex partial status epilepticus
Epilepsy	G418	Other status epilepticus
Epilepsy	G419	Status epilepticus unspecified
Acute nerve root/cord syndrome	G549	Nerve root and plexus disorder
Acute nerve root/cord syndrome	G551	Nerve root and plexus compressions in intervertebral disc disorders
Acute paralysis	G610	Guillain-Barre syndrome
Acute nerve root/cord syndrome	G820	Flaccid paraplegia
Acute nerve root/cord syndrome	G821	Spastic paraplegia
Acute nerve root/cord syndrome	G822	Paraplegia unspecified
Acute nerve root/cord syndrome	G823	Flaccid tetraplegia
Acute nerve root/cord syndrome	G824	Spastic tetraplegia
Acute nerve root/cord syndrome	G825	Tetraplegia unspecified
Acute nerve root/cord syndrome	G952	Cord compression unspecified
Acute nerve root/cord syndrome	G992	Myelopathy in diseases classified elsewhere
Subarachnoid haemorrhage	I600	Subarachnoid haemorrhage from carotid siphon and bifurcation
Subarachnoid haemorrhage	I601	Subarachnoid haemorrhage from middle cerebral artery

For peer review only

Subarachnoid haemorrhage	I602	Subarachnoid haemorrhage from anterior communicating artery
Subarachnoid haemorrhage	I603	Subarachnoid haemorrhage from posterior communicating artery
Subarachnoid haemorrhage	I604	Subarachnoid haemorrhage from basilar artery
Subarachnoid haemorrhage	I605	Subarachnoid haemorrhage from vertebral artery
Subarachnoid haemorrhage	I606	Subarachnoid haemorrhage from other intracranial arteries
Subarachnoid haemorrhage	I607	Subarachnoid haemorrhage from intracranial artery, unspecified
Subarachnoid haemorrhage	I608	Other subarachnoid haemorrhage
Subarachnoid haemorrhage	I609	Subarachnoid haemorrhage, unspecified
Subarachnoid haemorrhage	I690	Sequelae of subarachnoid haemorrhage
Acute nerve root/cord syndrome	M470	Anterior spinal and vertebral artery compression syndromes
Acute nerve root/cord syndrome	M471	Other spondylosis with myelopathy
Acute nerve root/cord syndrome	M490	Tuberculosis of spine
Acute nerve root/cord syndrome	M500	Cervical disc disorder with myelopathy - linked to G992A
Acute nerve root/cord syndrome	M501	Cervical disc disorder with radiculopathy
Acute nerve root/cord syndrome	M510	Lumbar and other intervertebral disc disorders with myelopathy
Acute nerve root/cord syndrome	M511	M51.1(dagger) Lumbar and other intervertebral disc disorders with radiculopathy (G55.1(asterix))
Acute nerve root/cord syndrome	M612	Paralytic calcification and ossification of muscle (Myositis ossificans associated with quadriplegia or paraplegia)
Head Injuries	S040	Injury of optic nerve and pathways

For peer review only - http://bmjopen.bmj.com/site/about/guidelines.xhtml

Head Injuries	S041	Injury of oculomotor nerve
Head Injuries	S042	Injury of trochlear nerve
Head Injuries	S043	Injury of trigeminal nerve
Head Injuries	S044	Injury of abducent nerve
Head Injuries	S045	Injury of facial nerve
Head Injuries	S046	Injury of acoustic nerve
Head Injuries	S047	Injury of accessory nerve
Head Injuries	S048	Injury of other cranial nerves
Head Injuries	S049	Injury of unspecified cranial nerve
Head Injuries	S060	Concussion
Head Injuries	S061	Traumatic cerebral oedema
Head Injuries	S062	Diffuse brain injury
Head Injuries	S063	Focal brain injury
Head Injuries	S064	Epidural haemorrhage
Head Injuries	S065	Traumatic subdural haemorrhage
Subarachnoid haemorrhage	S066	Traumatic subarachnoid haemorrhage
Head Injuries	S067	Intracranial injury with prolonged coma
Head Injuries	S068	Other intracranial injuries
Head Injuries	S069	Intracranial injury unspecified
Acute nerve root/cord syndrome	T093	Injury of spinal cord level unspecified

For peer review only

The RECORD statement – checklist of items, extended from the STROBE statement, that should be reported in observational studies using routinely collected health data.

	Item No.	STROBE items	Location in manuscript where items are reported	RECORD items	Location in manuscript where items are reported
**Title and abstract**					
	1	(a) Indicate the study's design with a commonly used term in the title or the abstract (b) Provide in the abstract an informative and balanced summary of what was done and what was found	Abstract, p2	RECORD 1.1: The type of data used should be specified in the title or abstract. When possible, the name of the databases used should be included.<br><br>RECORD 1.2: If applicable, the geographic region and timeframe within which the study took place should be reported in the title or abstract.<br><br>RECORD 1.3: If linkage between databases was conducted for the study, this should be clearly stated in the title or abstract.	Abstract, p2<br><br>Abstract, p2<br><br>Not applicable
**Introduction**					
Background rationale	2	Explain the scientific background and rationale for the investigation being reported	Introduction, p3		
Objectives	3	State specific objectives, including any prespecified hypotheses	Introduction, p3		
**Methods**					
Study Design	4	Present key elements of study design early in the paper	Methods, pp4-5		
Setting	5	Describe the setting, locations, and relevant dates, including periods of recruitment, exposure, follow-up, and data collection	Methods, pp4-5		

For peer review only

Participants	6	*(a) Cohort study* - Give the eligibility criteria, and the sources and methods of selection of participants. Describe methods of follow-up<br>*Case-control study* - Give the eligibility criteria, and the sources and methods of case ascertainment and control selection. Give the rationale for the choice of cases and controls<br>*Cross-sectional study* - Give the eligibility criteria, and the sources and methods of selection of participants<br><br>*(b) Cohort study* - For matched studies, give matching criteria and number of exposed and unexposed<br>*Case-control study* - For matched studies, give matching criteria and the number of controls per case	Retrospective empirical research based on routine healthcare data	RECORD 6.1: The methods of study population selection (such as codes or algorithms used to identify subjects) should be listed in detail. If this is not possible, an explanation should be provided.<br><br>RECORD 6.2: Any validation studies of the codes or algorithms used to select the population should be referenced. If validation was conducted for this study and not published elsewhere, detailed methods and results should be provided.<br><br>RECORD 6.3: If the study involved linkage of databases, consider use of a flow diagram or other graphical display to demonstrate the data linkage process, including the number of individuals with linked data at each stage.	Methods, pp4-5<br><br>Methods, pp4-5<br><br>Not applicable
Variables	7	Clearly define all outcomes, exposures, predictors, potential confounders, and effect modifiers. Give diagnostic criteria, if applicable.	Methods, pp4-5 and appendix 1	RECORD 7.1: A complete list of codes and algorithms used to classify exposures, outcomes, confounders, and effect modifiers should be provided. If these cannot be reported, an explanation should be provided.	Appendix 1
Data sources/ measurement	8	For each variable of interest, give sources of data and details of methods of assessment (measurement).<br>Describe comparability of assessment methods if there is more than one group	Methods, pp4-5		

For peer review only

Bias	9	Describe any efforts to address potential sources of bias	Methods, pp4-5		
Study size	10	Explain how the study size was arrived at	Methods, pp4-5		
Quantitative variables	11	Explain how quantitative variables were handled in the analyses. If applicable, describe which groupings were chosen, and why	Methods, pp4-5		
Statistical methods	12	(a) Describe all statistical methods, including those used to control for confounding<br>(b) Describe any methods used to examine subgroups and interactions<br>(c) Explain how missing data were addressed<br>(d) *Cohort study* - If applicable, explain how loss to follow-up was addressed<br>*Case-control study* - If applicable, explain how matching of cases and controls was addressed<br>*Cross-sectional study* - If applicable, describe analytical methods taking account of sampling strategy<br>(e) Describe any sensitivity analyses	Methods, pp4-5		
Data access and cleaning methods		..		RECORD 12.1: Authors should describe the extent to which the investigators had access to the database population used to create the study population.	Methods, pp4-5<br><br>Methods, pp4-5

For peer review only

				RECORD 12.2: Authors should provide information on the data cleaning methods used in the study.	
Linkage		..		RECORD 12.3: State whether the study included person-level, institutional-level, or other data linkage across two or more databases. The methods of linkage and methods of linkage quality evaluation should be provided.	Not applicable
**Results**					
Participants	13	(a) Report the numbers of individuals at each stage of the study (*e.g.*, numbers potentially eligible, examined for eligibility, confirmed eligible, included in the study, completing follow-up, and analysed)<br>(b) Give reasons for non-participation at each stage.<br>(c) Consider use of a flow diagram	Results, pp6-9	RECORD 13.1: Describe in detail the selection of the persons included in the study (*i.e.,* study population selection) including filtering based on data quality, data availability and linkage. The selection of included persons can be described in the text and/or by means of the study flow diagram.	Results, pp6-9
Descriptive data	14	(a) Give characteristics of study participants (*e.g.*, demographic, clinical, social) and information on exposures and potential confounders<br>(b) Indicate the number of participants with missing data for each variable of interest<br>(c) *Cohort study* - summarise follow-up time (*e.g.*, average and total amount)	Results, pp6-9		
Outcome data	15	*Cohort study* - Report numbers of outcome events or summary measures over time<br>*Case-control study* - Report numbers in each exposure	Results, pp6-9		

For peer review only

		category, or summary measures of exposure *Cross-sectional study* - Report numbers of outcome events or summary measures			
Main results	16	(a) Give unadjusted estimates and, if applicable, confounder-adjusted estimates and their precision (e.g., 95% confidence interval). Make clear which confounders were adjusted for and why they were included (b) Report category boundaries when continuous variables were categorized (c) If relevant, consider translating estimates of relative risk into absolute risk for a meaningful time period	Results, pp6-9		
Other analyses	17	Report other analyses done—e.g., analyses of subgroups and interactions, and sensitivity analyses	Results, pp6-9		
**Discussion**					
Key results	18	Summarise key results with reference to study objectives	Discussion, pp10-11		
Limitations	19	Discuss limitations of the study, taking into account sources of potential bias or imprecision. Discuss both direction and magnitude of any potential bias	Discussion, pp10-11	RECORD 19.1: Discuss the implications of using data that were not created or collected to answer the specific research question(s). Include discussion of misclassification bias, unmeasured confounding, missing data, and changing eligibility over time, as they pertain to the study being reported.	Discussion, pp10-11
Interpretation	20	Give a cautious overall interpretation of results considering objectives,	Discussion, pp10-11		

For peer review only

		limitations, multiplicity of analyses, results from similar studies, and other relevant evidence			
Generalisability	21	Discuss the generalisability (external validity) of the study results	Discussion, pp10-11		
**Other Information**					
Funding	22	Give the source of funding and the role of the funders for the present study and, if applicable, for the original study on which the present article is based	No study-specific funding		
Accessibility of protocol, raw data, and programming code		..	Methods, pp4-5 and appendix 1	RECORD 22.1: Authors should provide information on how to access any supplemental information such as the study protocol, raw data, or programming code.	Corresponding author details

*Reference: Benchimol EI, Smeeth L, Guttmann A, Harron K, Moher D, Petersen I, Sørensen HT, von Elm E, Langan SM, the RECORD Working Committee. The REporting of studies Conducted using Observational Routinely-collected health Data (RECORD) Statement. *PLoS Medicine* 2015; in press.

*Checklist is protected under Creative Commons Attribution (CC BY) license.

For peer review only
